# Bioinks and bioprinting technologies to make heterogeneous and biomimetic tissue constructs

**DOI:** 10.1016/j.mtbio.2019.100008

**Published:** 2019-05-25

**Authors:** N. Ashammakhi, S. Ahadian, C. Xu, H. Montazerian, H. Ko, R. Nasiri, N. Barros, A. Khademhosseini

**Affiliations:** a Center for Minimally Invasive Therapeutics (C-MIT), University of California – Los Angeles, Los Angeles, CA, 90095, USA; b Department of Bioengineering, University of California – Los Angeles, Los Angeles, CA, 90095, USA; c Division of Plastic Surgery, Department of Surgery, Oulu University, Oulu, 8000, Finland; d School of Dentistry, The University of Queensland, Herston, QLD, 4006, Australia; e Department of Mechanical Engineering, Sharif University of Technology, Tehran, 11365-11155, Iran; f Department of Radiological Sciences, University of California – Los Angeles, Los Angeles, CA, 90095, USA; g Department of Chemical and Biomolecular Engineering, University of California – Los Angeles, Los Angeles, CA, 90095, USA

**Keywords:** Three-dimensional bioprinting, Biofabrication, Regenerative medicine, Tissue engineering

## Abstract

The native tissues are complex structures consisting of different cell types, extracellular matrix materials, and biomolecules. Traditional tissue engineering strategies have not been able to fully reproduce biomimetic and heterogeneous tissue constructs because of the lack of appropriate biomaterials and technologies. However, recently developed three-dimensional bioprinting techniques can be leveraged to produce biomimetic and complex tissue structures. To achieve this, multicomponent bioinks composed of multiple biomaterials (natural, synthetic, or hybrid natural-synthetic biomaterials), different types of cells, and soluble factors have been developed. In addition, advanced bioprinting technologies have enabled us to print multimaterial bioinks with spatial and microscale resolution in a rapid and continuous manner, aiming to reproduce the complex architecture of the native tissues. This review highlights important advances in heterogeneous bioinks and bioprinting technologies to fabricate biomimetic tissue constructs. Opportunities and challenges to further accelerate this research area are also described.

## 1 Introduction

To engineer tissues for reconstructive surgical purposes, artificial matrices are conventionally fabricated and subsequently seeded with cells. However, this method is cumbersome, and it is associated with inhomogeneous cell distribution within the scaffolds and consequent problems with engineered tissue integration and remodeling [1]. Although several types of tissue-like constructs were successfully fabricated using three-dimensional (3D) bioprinting [2], [3], they are relatively simple, having one or two types of cells and usually consist of single or two materials/phases. To develop functional and biomimetic tissue-like constructs, it is important to consider different development stages that engineered tissues often need to go through, including cell viability and function *in vitro*, implantation, integration, and remodeling *in vivo*. To this end, tissue scaffolds should be cell-friendly, capable of maintaining cell viability, and can produce structures that can preserve their shape and mechanical properties for a period enough for tissue remodeling to take place and regenerated tissue to take over. So far, this was largely difficult to achieve because of the lack of necessary cellular or structural components in engineered constructs [4]. The native tissues possess more complex structures than engineered constructs and are composed of diverse types of materials, different types of parenchymal, stromal, and other types of cells as well as various biomolecules. In addition, extracellular matrix (ECM) is organized in a highly delicate manner to suit the specific function to be carried out by the tissue and organ [5]. The lack of appropriate control over spatial organization and components of commonly developed tissues is largely responsible for limited capability to produce biomimetic complex structures.

To solve this problem, the technology of 3D printing provides an attractive solution by employing multicomponent bioinks, which are by definition the materials that can be used for biofabrication [6], high resolution, and complex fabrication approaches can be employed to bioprint tissue constructs. 3D bioprinting has opened new avenues in mimicking the heterogeneous and complex native tissues. Bioprinting is basically a subset of biofabrication technologies [7]. In essence, the term ‘biofabrication’ refers to the products in which living cells, biomaterials, bioactive molecules, and cell aggregates are assembled through either bioprinting or bioassembly [8]. Heterogeneous bioinks are referred to those bioinks used in bioprinting wherein multiple cell types as well as biomaterials are present and function to mimic the target tissue [9]. With the advent of 3D printing, it is becoming possible to circumvent this problem and produce cell-laden tissue constructs with controlled architecture and resolution [10]. 3D bioprinting allows precise positioning of biomaterials and living cells layer by layer to fabricate 3D functional structures [11]. The ultimate aim of bioprinting is to develop 3D living human constructs with biological and physical properties that can match the native tissues, which would be able to repair tissue defects and restore organ structure and function.

Additive manufacturing technology was brought into the field of medical applications in early 2000 [12], [13]. In early stages of development, a single biomaterial or simply physical mix of individual materials and a single cell type were used as bioinks for 3D bioprinting techniques. Concurrent with the advance of additive manufacturing techniques, 3D printers were rapidly tailored for fabricating bone tissue [14], blood vessels [15], and simple organs such as skin [16], [17]. Multiple techniques have been recently developed to solve this obstacle and achieve bioprinting multimaterials spontaneously in a continuous manner [18], aiming to reproduce the complex microarchitecture of the native tissues with varying types of biomaterials and cells. These methods are primarily classified as extrusion, inkjet, laser-assisted, and UV-assisted bioprinting [19].

**Fig. 1:**
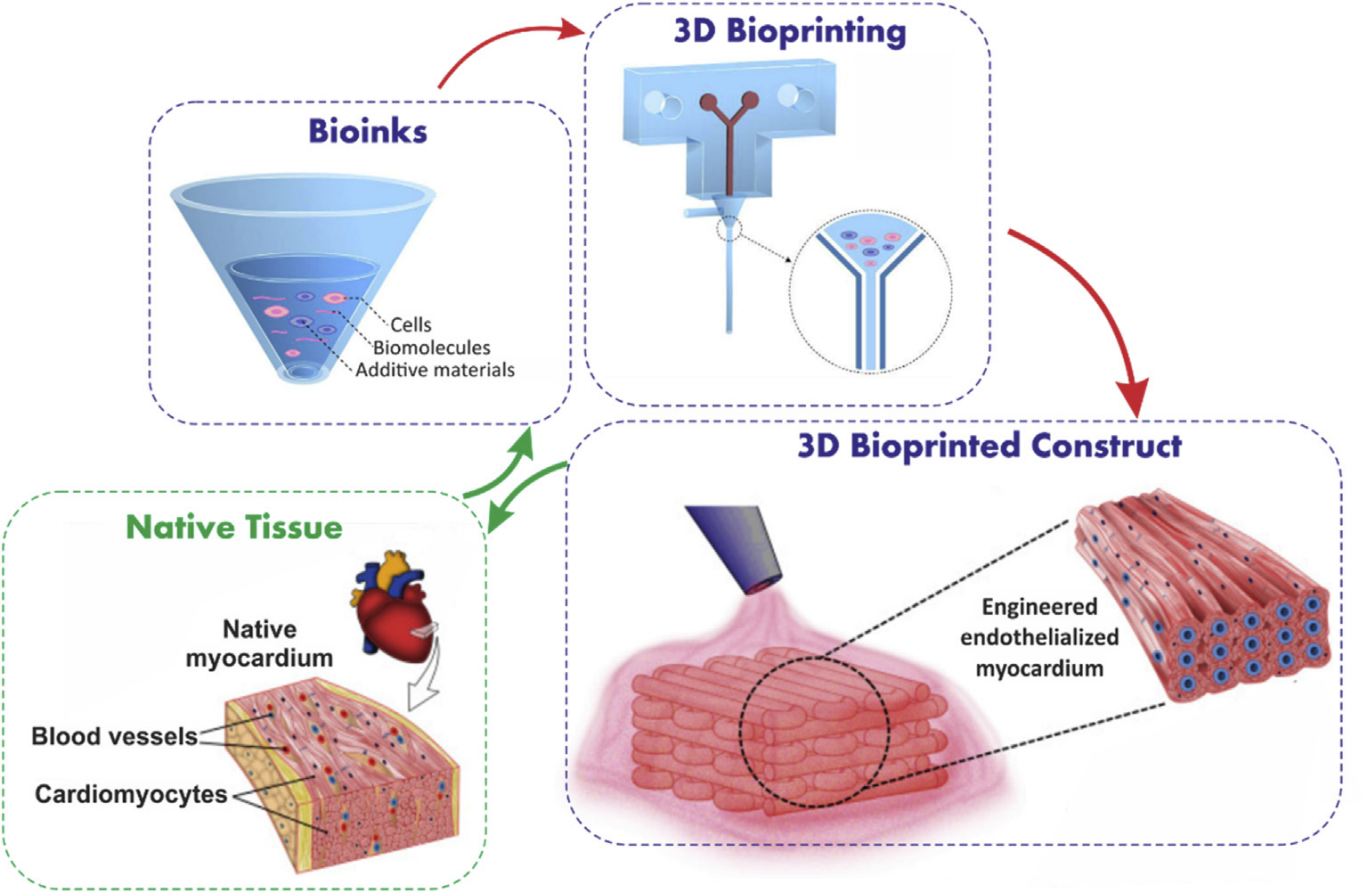
Schematic representation of the procedure for design and biofabrication of tissue constructs from bioinks mimicking the native tissue parts of figure are reproduced from Zhang et al. [20] with permission from Elsevier.

The present review highlights important advances in the development of multicomponent bioinks and the emerging bioprinting techniques toward ​developing biomimetic tissue-like constructs. The present review focuses mainly on the direct printing approaches wherein multicomponent and heterogeneous bioinks are fabricated in one-step manner. We also discuss reported examples of bioprinted functional tissues. Eventually, challenges and future perspectives for better mimicking the complexity and function of the native tissues using 3D bioprinting approaches are described (see Fig. 1).

## 2 Multicomponent bioinks

Bioinks are referred to as cell-laden fluid materials that may have additional containing matrix components, and they are loaded into the 3D printers for fabricating tissue-like constructs [21]. Multicomponent bioinks are defined as a mix of more than one type of biomaterial, one or more than one type of cells, and additive materials or biomolecules. Several multicomponent bioinks have been developed, and they are referred to as multimaterials [22] or multicellular bioinks [23]. Various biomaterials have been used in these bioinks for building different tissue constructs [10], [11]. For example, natural polymers, such as collagen and gelatin, have been widely used because they contain Arg-Gly-Asp (RGD) motifs [24], which are important for cell attachment and migration. However, these materials often suffer from low mechanical properties, and thus, other biomaterials have been combined, and additive elements are employed to obtain multimaterial bioinks with improved properties as compared with conventional single material-based bioinks [1]. Resulting multicomponent bioinks should have chemical and physical properties that can allow their printability [25]. In general, rheological properties of multicomponent bioinks should be precisely controlled to achieve printability and structural stability of bioprinted structures. In addition to varying bioink types, it is also possible to vary properties of the material in a given bioink to achieve a bioink with diverse properties, such as producing bioinks with varying degrees of stiffness [26]. Multicomponent bioinks should also be crosslinkable or can solidify after bioprinting to produce stable 3D bioprinted constructs. The bioink should neither solidify too quickly, leading to clogging of the printer nozzles nor too slowly leading to the collapse of bioprinted construct because of the effect of building up weight of bioprinted layers [27]. Biomaterials constituting bioink should be degradable yet retain enough mechanical properties for a period of time sufficient to support tissue regeneration and subsequent tissue remodeling and maturation [28]. Such multimaterial combination should not be toxic to cells or tissues (materials themselves, their degradation products, metabolites, or possible interactions at cellular level), in short or long-term [29].

**Fig. 2:**
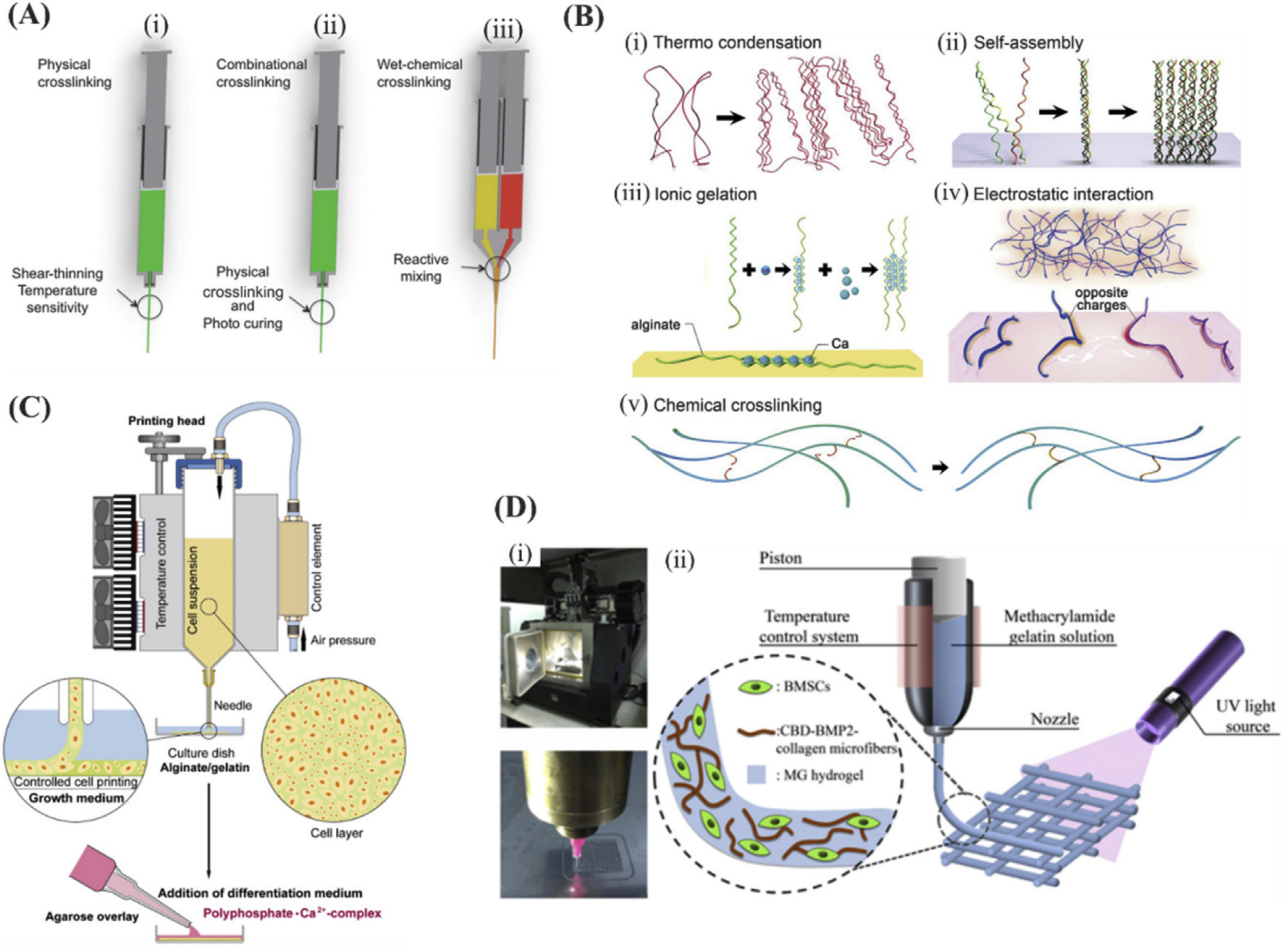
Different crosslinking methods of bioinks. (A) Schematic of crosslinking approaches including ​(i-ii) physical, combinational, and (iii) wet-chemical crosslinking approaches in extrusion printing. Reproduced from Malda et al. [37] with permission from Wiley-VCH Verlag GmbH & Co. (B) Hydrogel crosslinking approaches. (i to iv) Physical crosslinking approaches including: (i) thermally induced polymer chain entanglement, (ii) molecular self-assembly, (iii) ionic gelation, (iv) electrostatic interaction, and (v) chemical crosslinking. (Scale bar ​= ​100 ​μm). Reprinted from Zhang and Khademhosseini [31], with permission from AAAS. (C) Schematic indicating 3D cell bioprinting of SaOS-2 ​cells in gelatin/alginate. The bioprinted bioink was then put in a calcium chloride (CaCl_2_) solution. This construct was then covered with an agarose layer and cultured in osteogenic differentiation medium. Reproduced from Neufurth et al. [38] with permission from Elsevier. (D) Schematic indicating the construct photocrosslinking. Reproduced from Du et al. [39] with permission from IOP. BMSCs, bone marrow–derived mesenchymal stem cells; BMP2, bone morphogenetic protein 2.

In general, biomaterials used in bioinks can be classified into shear-thinning and non–shear-thinning materials. Shear-thinning materials can be injectable under the application of shear force and have the ability to quickly self-heal after removal of the shear force [30]. For non–shear-thinning materials, gelation occurs under the influence of physical or chemical stimuli. Physical crosslinking includes thermocondensation, self-assembly, ionic gelation, or electrostatic interaction. Chemical crosslinking can be achieved by the formation of covalent bonds between chains of a macromolecule under appropriate circumstances [31]. Common examples of physical crosslinking include the use of calcium ions for crosslinking alginate [32], and common example for chemical methods include the use of UV light for crosslinking gelatin methacryloyl (GelMA) hydrogels [33] (Fig. 2).

Dynamics of the ECM is one important aspect of multicomponent bioink design. Reversible changes in hydrogels as response to stimuli, such as pH, light, magnetic, and electric field, as well as temperature can be exploited to recapitulate such dynamic characteristics required for cell spreading, matrix mechanics as well as biochemical ligand presentation [34]. In this context, viscoelastic characteristics of hydrogels is one of the main time-dependent factors that should be tailored based on the native tissue. Shear-thinning biomaterials are of high interest in this regard because they reform once the shear stress is removed upon the material [30]. Other examples of dynamically crosslinked networks involve crosslinked Ag nanoparticles and hyaluronan (HA) hydrogels in which crosslinking increases with increasing thiolate concentration [35]. The dynamic mechanical loading was reported to affect the chondrocyte gene expression in polyethylene glycol (PEG)-RGD hydrogels [36]. In the latter study, it was stipulated that mechanical stimulation improved the cartilage-specific gene expression.

### 2.1 Multicomponent bioinks

A single biomaterial in bioinks cannot usually meet all mechanical and functional requirements, which are essential to produce biomimetic tissue-like constructs. The use of biomaterials, such as PEG, allows the control via varying molecular weight and crosslinking of physical properties of the resulting construct. However, it lacks biological cues necessary for cell adhesion, proliferation, and spreading [40], [41]. On the other hand, cell-friendly and natural biomaterials, such as gelatin and fibrin, are limited by their poor mechanical properties [42], [43]. Thus, multicomponent bioinks comprised of more than one material may combine favorable properties of individual materials. These bioinks have become more attractive for 3D bioprinting of constructs with improved performance. In this section, multicomponent bioinks are categorized and discussed based on their constitutive materials. Moreover, methods to combine different biomaterials, including simple mixing, postcoating, and chemical crosslinking together with advantages and limitations associated with each method are detailed [31], [44].

#### 2.1.1 Bioinks having combination of natural materials

Hydrogels have commonly been used for bioprinting owing to their biocompatibility and high water content [45]. A suitable hydrogel for bioprinting should have a storage modulus in the order of 10^2^–10^3^ ​Pa. Otherwise, the hydrogel would be either too fluidic or too stiff to achieve effective 3D printing [3], [5]. In general, hydrogels with a viscosity of 30 ​mPa ​s to >6 ​× ​10^7^ ​mPa ​s are suitable for extrusion 3D bioprinting [5]. However, hydrogels do not generally have enough strength to enable engineering of mechanically demanding tissues, such as bone, cartilage, and tendon. Thus, it is needed to adjust the strength of hydrogels by combining them with other biomaterials to obtain functional multicomponent-based bioinks.

##### 2.1.1.1 Alginate with gelatin/fibrin

**Fig. 3:**
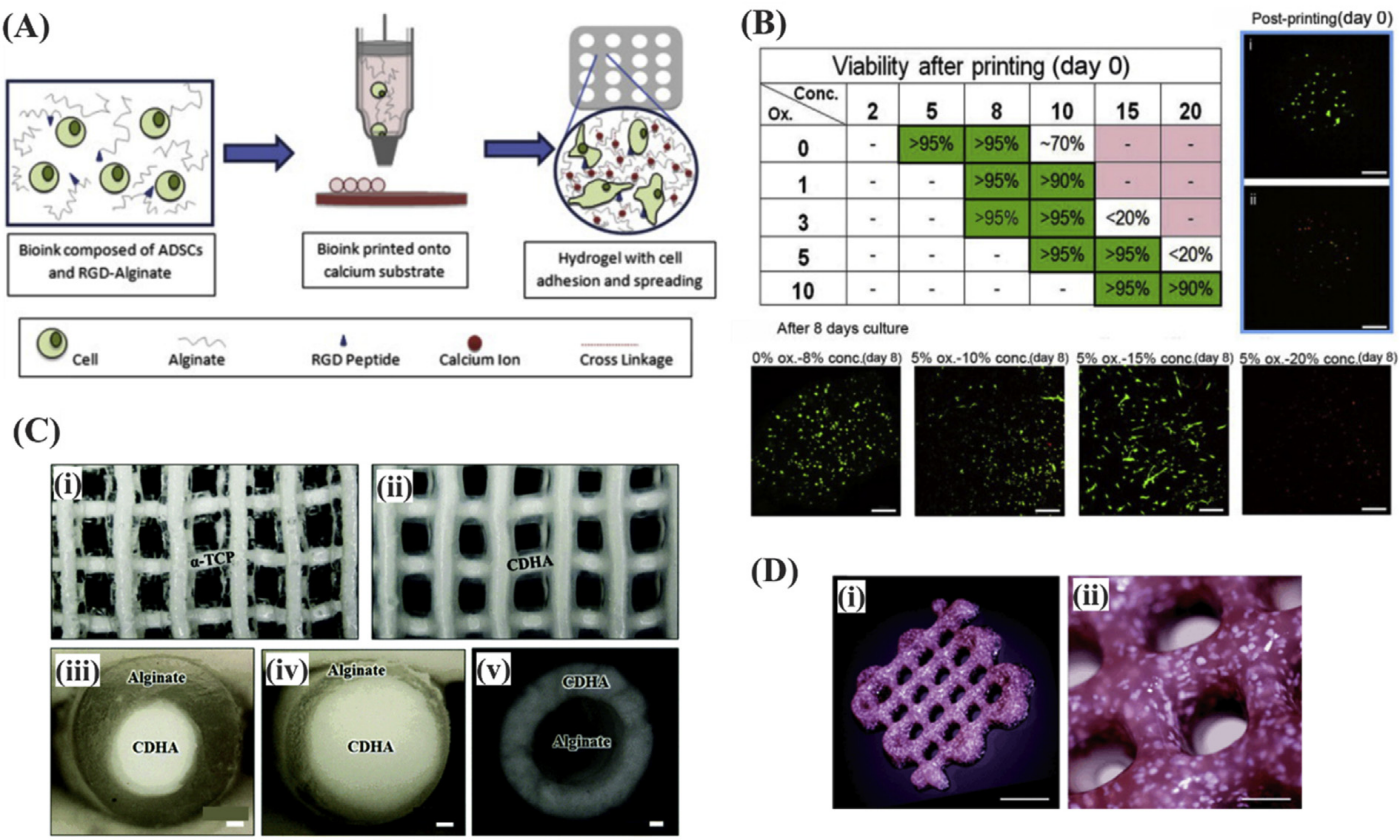
(A) Schematic illustration of droplet-based fabrication process for lattice structure made of human adipose tissue–derived stem cells encapsulated in alginate and (B) the resulting cell viability as a function of bioink parameters. Alginates with medium viscosity resulted in higher cell viability owing to limited nutrition transfer at high and low concentrations. Reproduced from Jia et al. [48] with permission from Elsevier. (C) Representation of 3D printed alpha tricalcium phosphate (α-TCP)/alginate core/shell scaffolds (i) before and (ii) after crosslinking, (iii-iv) fiber cross sections demonstrating the core/shell structure of alginate and calcium-deficient HAp. Scale bar ​= ​100 ​μm. Reproduced from Raja et al. [49] with permission from the Royal Society of Chemistry. (D) Three-dimensional printing of a porous scaffold consisting of alginate, mesenchymal stem cells and, calcium phosphate particles using extrusion printing. Scale bar ​= ​500 ​μm. Reproduced from Loozen et al. [50] with permission from the Royal Society of Chemistry. Hap, hydroxyapatite; RGD, Arg-Gly-Asp.

Alginate has widely been used for bioprinting because of its high biocompatibility and rapid crosslinkability. Alginate is a natural, seaweed-derived, ion-sensitive, and anionic polysaccharide [38]. This hydrogel hardens by exposure to CaCl_2_ [46] as it leads to instantaneous formation of a gel via sodium-calcium ion exchange reaction occurring at ambient temperature [47]. Alginate provides a cell-protective effect against processing pressure stress, as it was demonstrated by resulting high cell viability rates following its use in 3D bioprinting (Fig. 3A and B) [38], [48]. Alginate was used for bioprinting of preosteoblast MC3T3-E1 cells in core-shell constructs (Fig. 3C) [49] ​and bone marrow–derived mesenchymal stem cells (BMSCs) with bone morphogenetic protein 2 (BMP-2) plasmid (Fig. 3D) [50]. Although alginate has similarities with the ECM glycosaminoglycans, it lacks bioactivity. Alternatively, gelatin (a desaturated collagen) has RGD sequence, and thus, it is a preferred biomaterial because of its enhanced cell attachment and function properties [51], [52]. In one study, Chung et al. bioprinted a combination of alginate and gelatin. The resulting structures exhibited mechanical properties similar to those of precrosslinked alginate and better cell growth [53]. Alginate was also combined with fibrin to improve the interaction of the bioink with cells [54]. Moreover, alginate can be combined with other biomaterials such as polyvinyl alcohol and hydroxyapatite (HAp) to produce multicomponent inks [55].

##### 2.1.1.2 Silk fibroin with gelatin

Silk fibroin is basically a protein produced by silkworm and contains a repeating pattern of Gly-Ser-Gly-Ala-Gly-Ala units [56]. Silk fibroin has superior mechanical properties and offers tunable degradability while gelatin has RGD sequences. Compared with silk fibroin alone, silk fibroin-gelatin scaffolds had balanced mechanical properties and degradation rates when used for cartilage regeneration [57]. Silk fibroin/gelatin was also used to develop bioinks for 3D bioprinting and delivery of human turbinate mesenchymal stromal cells (hTMSCs). The gelation was performed through enzymatic and physical crosslinking (through mushroom tyrosinase and sonication, respectively) [58]. In addition, the bioink was reported to positively regulate chondrogenic marker expression in the chondrocyte-laden constructs [59].

##### 2.1.1.3 Agarose with collagen

Agarose is a polysaccharide, which is derived from seaweed. It forms a gel at 34–38°C and melts at higher temperatures. Agarose is characterized by excellent gel formation property but it lacks the ability to support cell growth [60]. Agarose was used with collagen type I to confer mechanical support to bioprinted collagen constructs ​because collagen has low viscosity and slow gelation. Loaded cells in the bioinks were found to be viable until 21 days after culture indicating that the 3D bioprinting process did not adversely affect the cell viability [61].

##### 2.1.1.4 Chitosan with gelatin

Chitosan is an attractive alkaline polysaccharide biomaterial because it is biocompatible, biodegradable, and has antimicrobial properties [62]. However, chitosan suffers from slow gelation and low mechanical properties [47]. When chitosan was compared with alginate hydrogels, chitosan was found to be better than alginate alone in terms of osteogenic cell proliferation and differentiation [63]. Because chitosan is positively charged, it can be mixed with negatively charged gelatin at pH of 6.5 to form polyelectrolyte complex, and the hybrid hydrogel has good printability at room temperature, high 3D construct shape fidelity, and good biocompatibility [63].

##### 2.1.1.5 Cellulose with alginate

**Fig. 4:**
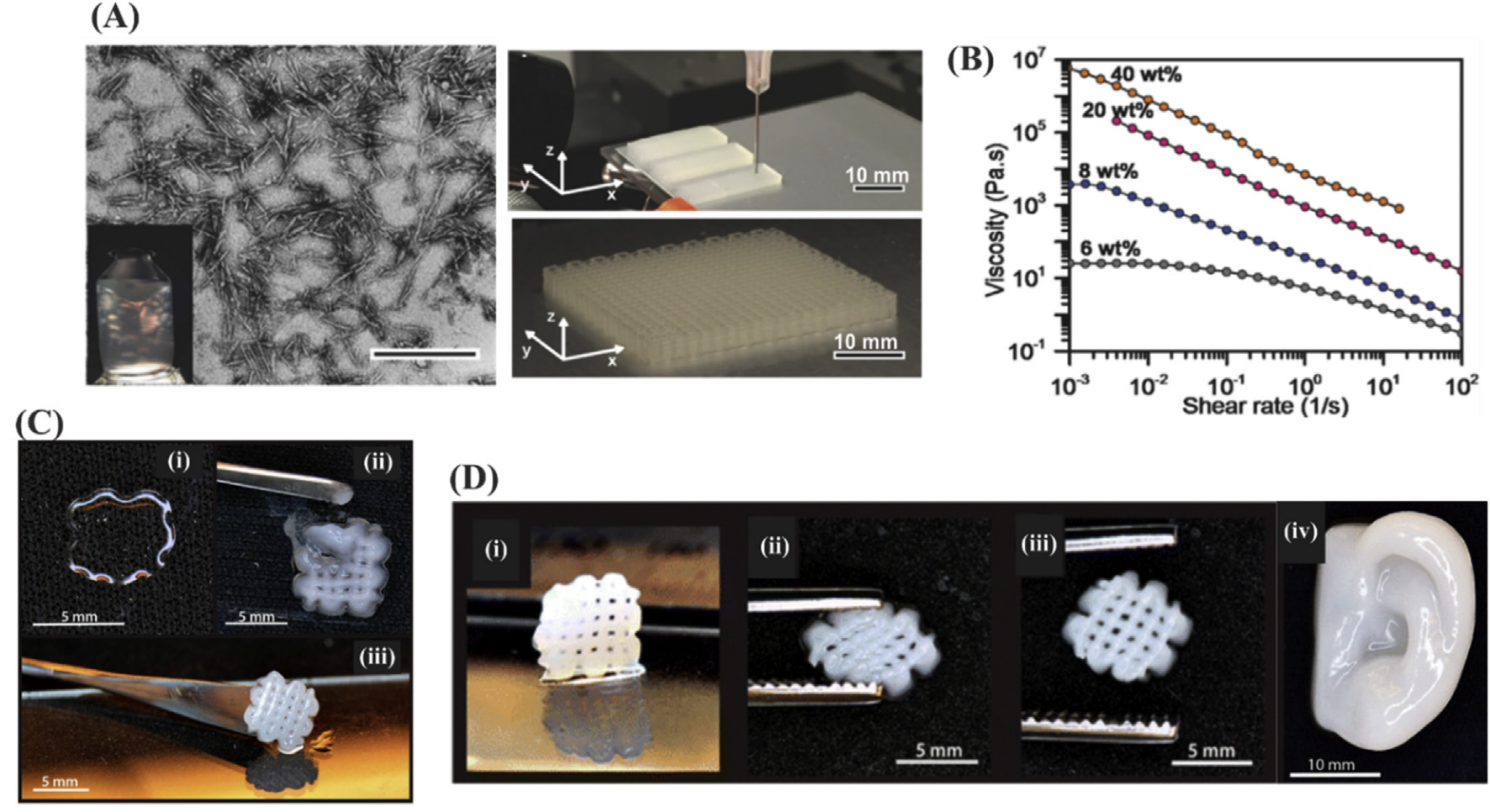
(A) Wood pulp CNC distribution in the aqueous inks (scale bar ​= ​500 ​nm) and photograph of the printed cellular constructs based on CNC inks. (B) Shear-thinning was induced to the ink slightly at 1% CNC concentration and increased significantly at around 10–20% as the viscosity decreased with shear rate. Reproduced from Siqueira et al. [65] with permission from Wiley-VCH Verlag GmbH & Co. (C) The printed constructs for a grid design obtained by using (i) 3% alginate, (ii) 2.5% NFC, or (iii) alginate/NFC inks. Combining the inks resulted in successful and structurally integrated (fully cured) constructs. (D) (i-iii) Mechanical recovery of the meshes and (iv) human ear model obtained by the use of alginate/NFC inks. Reprinted from Markstedt [67] with permission from the American Chemical Society. CNC, cellulose nanocrystals; NFC, nanofibrillated cellulose.

Cellulose is a linear polysaccharide composed of linked d-glucose units obtained from plants or bacteria [64]. Cellulose-based viscoelastic inks can be prepared by simply suspending cellulose nanocrystals (CNC) in water or photopolymerizable monomer solution to print porous architected constructs (Fig. 4(A)) [65]. Higher concentration of CNC (∼10–20%) was found to confer significant shear-thinning property to the ink (Fig. 4B) as viscosity decreased significantly when shear rate was increased from 0.01 to 50 ​s^−1^. The shear-thinning property of nanocellulose enabled the use of nanocellulose/alginate inks for printing of chondrocyte-laden bioinks into 3D constructs [66]. As it is represented in Fig. 4, 3D printing of alginate alone suffers from low printing fidelity because of low viscosity of the alginate ink (Fig. 4C [i]). On the other hand, nanofibrillated cellulose (NFC)–based inks were challenging in curability because the resulting grid structure could not be lifted up from the substrate (Fig. 4C [ii]). However, the inks comprised of both alginate and NFC resulted in successful 3D printing of structure allowing curing and shape fidelity (Fig. 4C [iii]). Optimal printing of the alginate/NFC inks was addressed in terms of high strain recovery and printing fidelity for 3D complex structures, such as human ear (Fig. 4(D)).

##### 2.1.1.6 Hyaluronan with cellulose

**Fig. 5:**
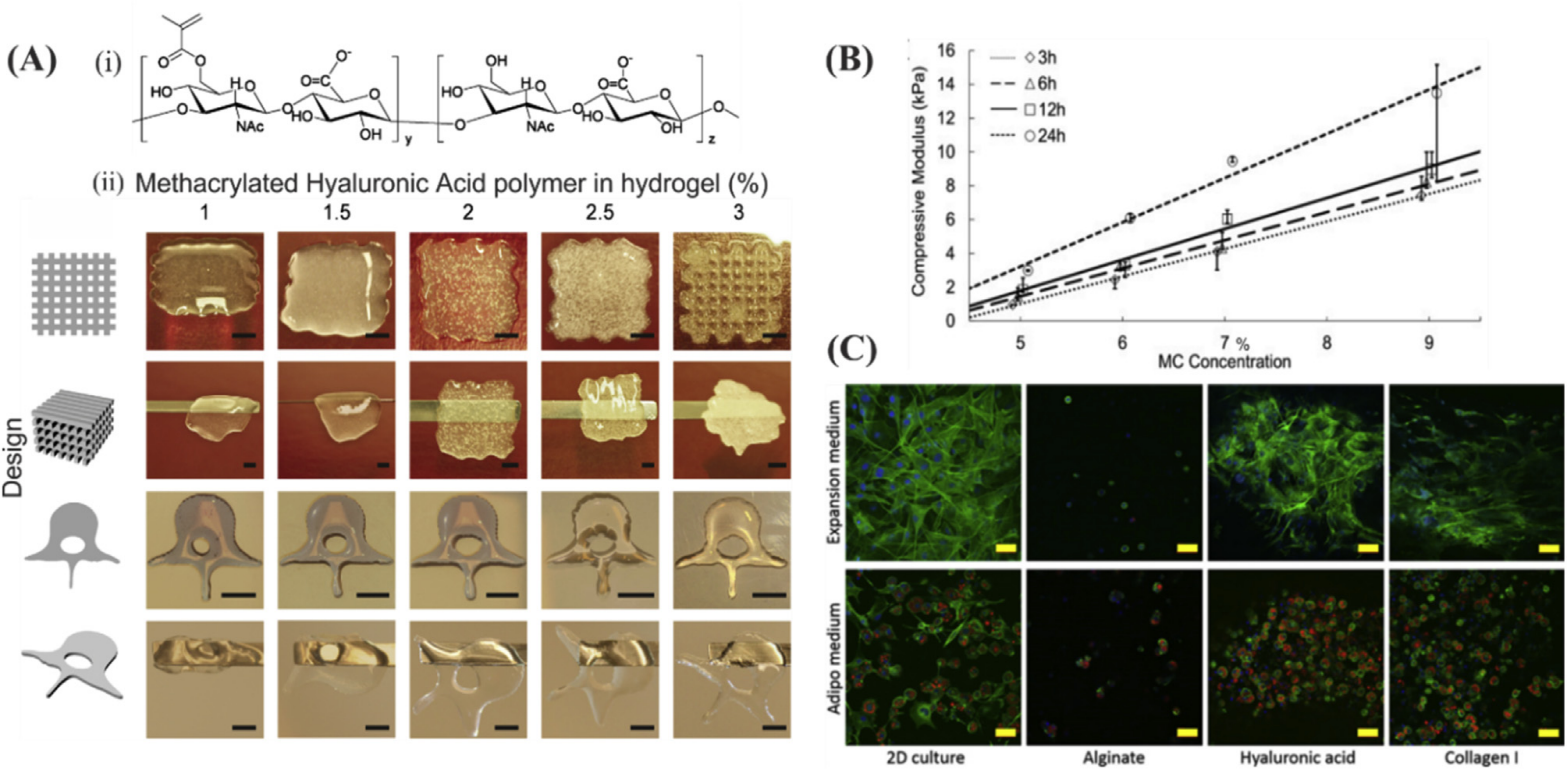
Characteristics of printed constructs based on HA modified inks. (A) (i) Chemical structure of methacrylated HA obtained by reacting HA with methacrylic anhydride in an aqueous environment and (ii) printability of methacrylated hyaluronic acid (MeHA) at different concentrations (scale bar ​= ​500 ​μm). The best printability was attained at MeHA concentration of 3%. Reproduced from Poldervaart et al. [71] (B) Compressive elastic properties of HA methylcellulose as a function of methylcellulose at different time points. Reproduced from Law et al. [74] with permission from Elsevier. (C) Comparing cell response in two different culture media (expansion and adipo), for cells seeded on either 2D surface or encapsulated in alginate, HA, or in collagen gels. Promoted cell differentiation and proliferation were seen in HA and collagen I gels (scale bar ​= ​50 ​μm; nuclei, actin filaments, and lipid droplets were shown in blue, green, and red, respectively). Reproduced from Henriksson et al. [75] with permission from IOP Publishing Copyright 2016. HA, hyaluronan.

HA is non-sulfated glycosaminoglycan that is found especially in connective, epithelial, and neural tissues [68]. It is also an important component of cartilage, which contributes to joint hydration and cell matrix interaction [69]. HA was also used for bioprinting [70], but its wide bioprinting application was limited by its low mechanical properties. One possibility to tackle this problem is by its methacrylation, which renders HA photocrosslinkable [71] and makes it resistant to degradation [72], [73]. Gels with high concentrations of methacrylated HA inks not only lead to better printability (Fig. 5A) ​but also to spontaneous osteogenic human BMSCs ​differentiation even without the use of other stimuli [71]. Furthermore, mechanical properties of HA hydrogels can be tuned in the range of ∼1–15 ​kPa by mixing with different concentrations of methylcellulose (Fig. 5B) [74]. When adipocyte-laden nanocellulose/HA bioinks were used for 3D bioprinting, cell viability was as high as 95% at 1 week postprinting [75]. After 2 weeks, the expression of adipogenic maker genes was much higher in 3D bioprinted constructs compared with two-dimensional (2D) cultured ones. Moreover, the results demonstrated that compared with alginate, HA and collagen both can promote MSC differentiation in adipogenic media and thereby they can form efficient bioinks in combination with nanocellulose for adipocyte cell culture (Fig. 5C).

#### 2.1.2 Bioinks comprising natural and synthetic components

Natural and synthetic polymers can be combined to obtain biomaterials with improved biocompatibility, mechanical performance, thermal properties, and crosslinkability. Many studies have used synthetic biomaterials in combination with natural ones to induce desired physical and chemical properties to the resulting composite, such as reinforcing the material or controlling shear-thinning properties. In the following sections, material compositions typically used in multicomponent bioinks are introduced.

**Fig. 6:**
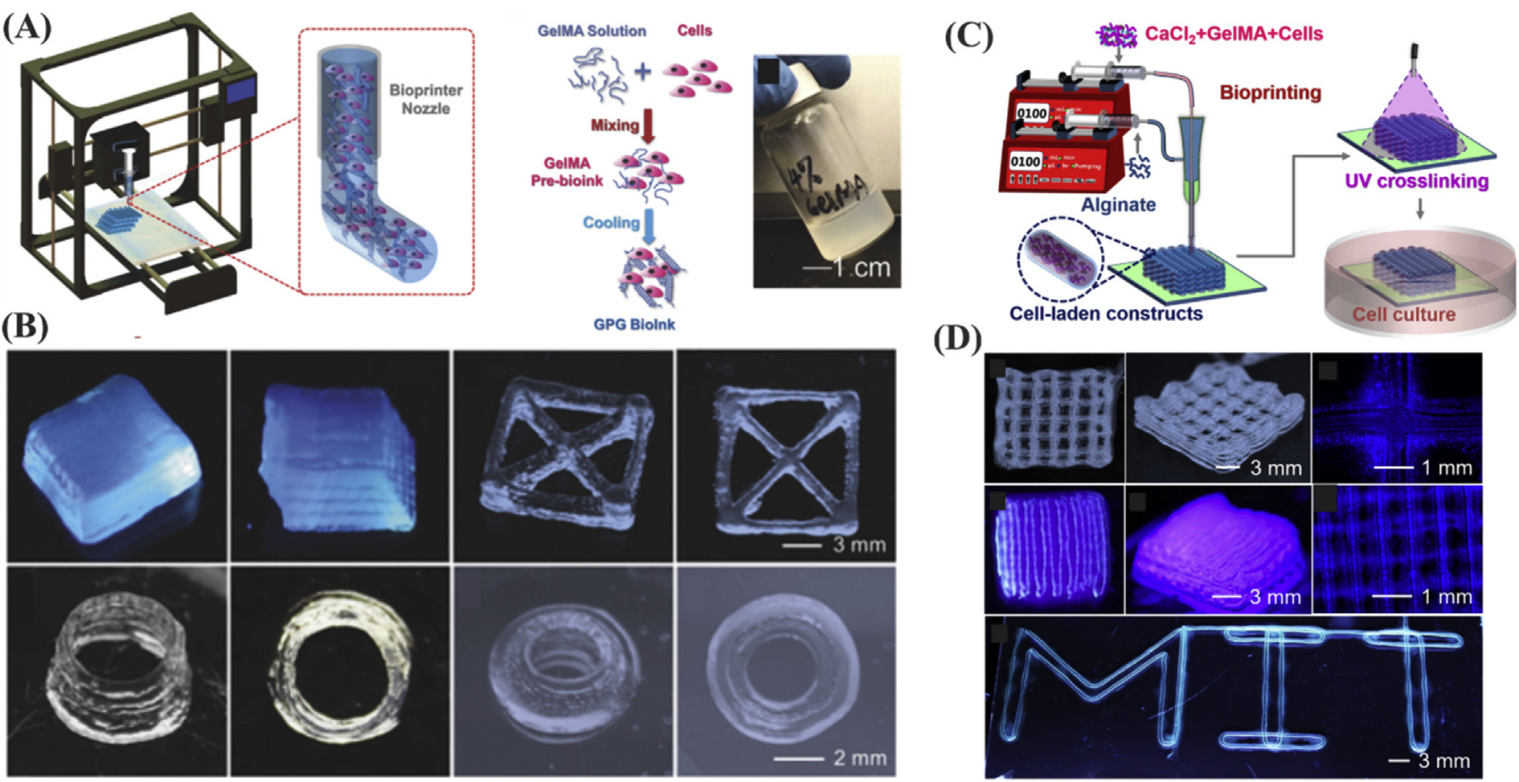
(A) 3D bioprinting of bioinks composed of cells and GelMA. (B) Low concentration 3D GelMA structures were fabricated by taking advantage of shear-thinning properties of GelMA, i.e. cooling down the structures to maintain their structural integrity. Reproduced from Liu et al. [78] with permission from WILEY-VCH Verlag GmbH & Co. (C) Illustration of GelMA/alginate microfibers with core/sheath architecture forming bioprinted constructs through extrusion 3D bioprinting. (D) Alginate sheath allows printing of 3D structures using low concentration GelMA (lower than 2%). Reproduced from Liu et al. [76] with permission from IOP Publishing. GelMA, gelatin methacryloyl.

GelMA is commonly used in bioinks [19], and it can provide favorable environment for cellular activities, including proliferation, spreading, migration, and differentiation [76]. GelMA is characterized by combining cell biocompatibility properties of gelatin with crosslinkability and mechanical strength conferred by methacryloyl component [3], [33], [77], which becomes an increasingly important biomaterial for 3D bioprinting [33], [77]. Bioprinted cell-laden GelMA is characterized by having high structural fidelity after deposition. Some hybrid GelMA constructs have become popular because of intrinsic shear-thinning and self-healing properties (Fig. 6A and B) [78]. In addition, it is possible to link growth factors, such as BMP-2 to GelMA binding domains for controlled BMP-2 release [39]. Although, 3D bioprinting using GelMA bioinks at low concentrations is favorable for cellular activity, it is a challenging process. To tackle this, Liu et al. [78] used alginate sheath as a template for low concentration GelMA bioinks. In this way, the alginate sheath provided mechanical support for core GelMA ink, while UV crosslinking occurred (Fig. 6C and D). In another study, physically crosslinkable alginate was employed as a temporal structural support to maintain the designed shape for GelMA during the bioprinting process. Then, the alginate could be removed selectively leaving behind the desired construct shape [79]. Mechanical strength, stability, and cell growth in GelMA/alginate hydrogels were also improved by adding 8 arm PEG acrylate with tripentaerythritol core [80]. Furthermore, the addition of gold nanorods was reported to promote synchronous electrical signal propagation in the hydrogels [81].

#### 2.1.3 Bioinks comprised of synthetic biomaterials

PEG is a linear polymer and it has been widely used in fabrication of various medical and pharmaceutical products. It is available in many chemical variants (linear or multi-arm) with different molecular weights [82]. PEG is water-soluble and pure PEG is not suitable for 3D bioprinting. The most common way of using PEG as bioink is by mixing it with poly(ethylene glycol) diacrylate (PEGDA) or methacrylate (PEGMA) [83], [84]. Although PEG is hydrophobic, it was found that cells such as osteoblasts can be encapsulated and survive well inside PEG biomaterials, such as PEGMA [85].

**Fig. 7:**
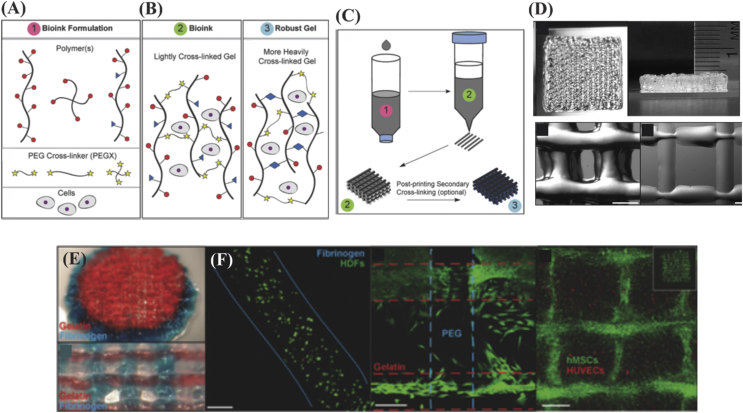
(A) Different types of polymer chain, crosslinker, and cells for developing cell-incorporated inks based on PEG. (B) Ink configuration after lightly and heavy crosslinking of PEG. (C) Three-dimensional printing strategy of developed PEG ink. Secondary crosslinking may be applied for heavily crosslinking the polymer chains after the 3D printing process was completed. (D) Photograph of 3D printed structures using PEG-gelatin bioinks (scale bar ​= ​500 ​μm). (E) Examples of combined 3D bioprinting of PEG-gelatin (red) and PEG-fibrinogen (blue) bioinks in spheroidal and grid designs and (F) cell viability results associated with using 3 w/v% fibrinogen in PEG, PEG-PEG, and PEG-gelatin (scale bar ​= ​200 ​μm). Human umbilical vein endothelial cells (HUVECs) seeded with human MSCs, which filled the pore spaces in the internal structure. Reproduced from Rutz et al. [87] with permission from WILEY-VCH Verlag GmbH & Co. PEG, polyethylene glycol; MSC, mesenchymal stem cell.

PEG has been mixed with other polymers to improve its mechanical properties. For example, when PEGDA was mixed with alginate, elastic modulus of the resulting hydrogel was increased from ∼5 to ∼75 ​kPa [86]. In addition, PEG is an ideal biomaterial for use as a linker for conjugating different polymers in ink. For example, Rutz et al. developed a method to produce multipolymer inks by using crosslinked PEG-modified polymers. First, they lightly crosslinked polymer network by a chemical crosslinking followed by a secondary crosslinking to manipulate elastic modulus and degradation properties. Furthermore, viscosity of these bioinks was tunable to enhance the print fidelity (Fig. 7) [87]. In another study, PEGMA was combined with bioactive glass (BaG) nanoparticles and HAp, and the mixture was used for human MSC bioprinting [88]. A high cell viability was observed along with higher compressive modulus of ∼358 ​kPa.

#### 2.1.4 Bioinks comprised of hydrogels and particles

**Fig. 8:**
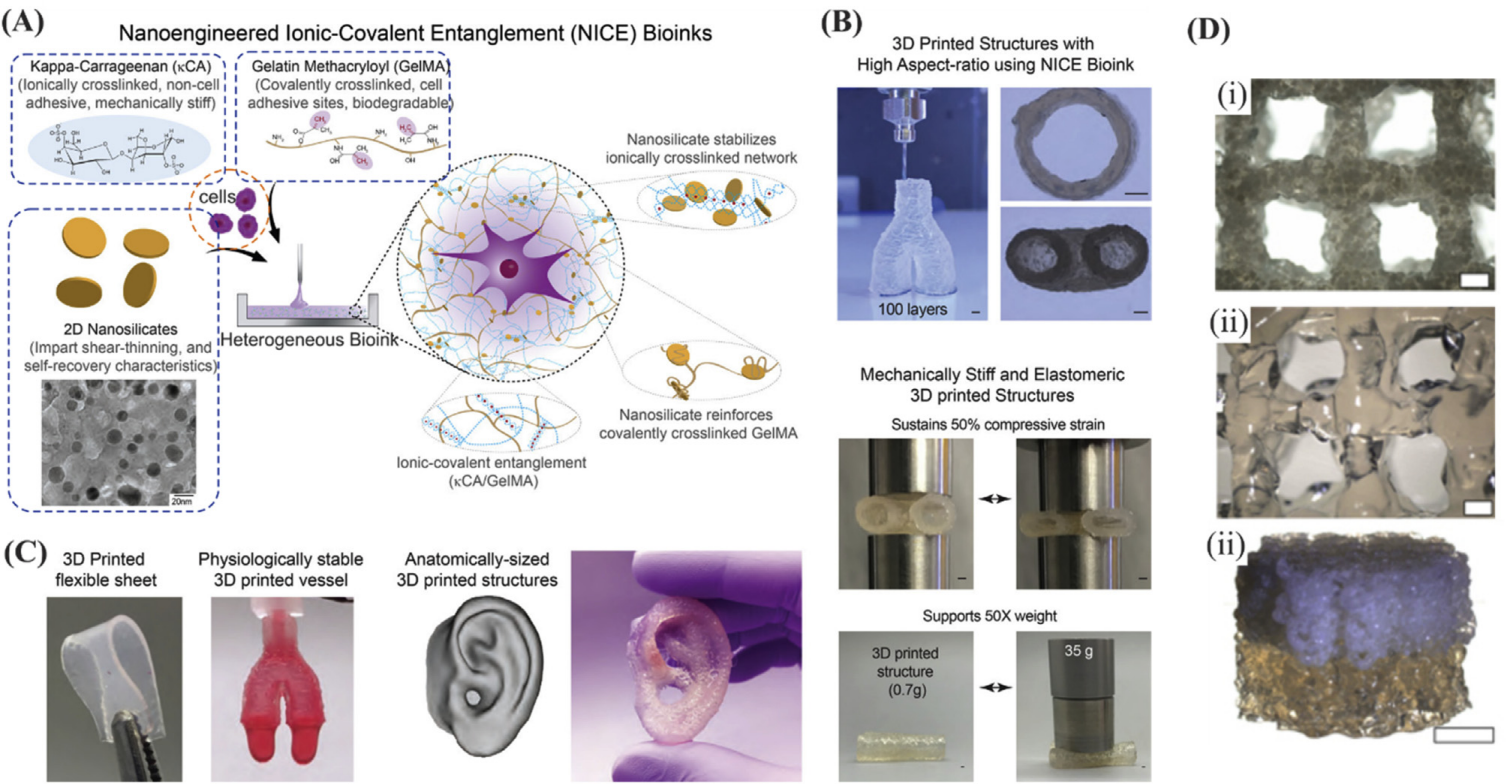
(A) Nanoengineered ionic-covalent entanglement (NICE) bioinks developed by taking advantage of nanoparticle ingredients: (1) Kappa-Carrageenan (κCA) for ionic crosslinking, (2) GelMA for covalent crosslinking, tissue adhesion, and biodegradability, and (3) 2D nanosilicates for having shear-thinning properties. (B) The NICE-based printed constructs exhibit promising mechanical recovery behavior (scale bar ​= ​1 ​mm). (C) High printing fidelity was achieved by the NICE ink because of versatile printing of complex 3D structures and human organs. Reprinted with permission from Ref. [92]. Copyright 2018 American Chemical Society. (D) Porous constructs fabricated by gelatin methacrylamide-gellan gum MSC-laden bioinks: (i) MSC-laden layer (scale bar ​= ​400 ​μm), (ii) GelMA-gellan gum layer (scale bar ​= ​400 ​μm), and (iii) perspective photograph of the bilayered GelMA-gellan gum cylindrical osteochondral graft model (scale bar ​= ​4 ​mm). Reproduced from Levato et al. [91] with permission from IOP Publishing. GelMA, gelatin methacryloyl, MSC, mesenchymal stem cell.

Mechanical properties of hydrogels can be dramatically improved by adding specific nanomaterials. Some nanomaterials can be used as crosslinkers to anchor polymer chains and improve the mechanical strength of hydrogels [89]. In general, there are many reports on using nanoparticles for tuning mechanical, chemical, and electrical properties of 3D bioprinted constructs. For instance, microparticles made of poly(lactide-*co*-glycolide)-PEG (PLGA-PEG) were used for improving mechanical properties of cell-laden carboxymethyl cellulose (CMC). Constructs having mechanical properties in the range matching those of cancellous bone were obtained (Young's modulus of 54.4–57.3 ​MPa and yield stress of 1.22–1.15 ​MPa) [90]. The PLGA-PEG microparticles were suspended in culture medium, with either CMC or Poloxamer 407 (Pluronic F-127) to increase its viscosity. When BMSCs were encapsulated and bioprinted, more viscous materials were associated with high mechanical properties. Unfortunately, higher content of microparticles adversely affected cell viability probably because of increased stress on the cells during the extrusion process (reduced lubricant carrier). Therefore, further work is needed to define balance between cytocompatibility and CMC/PLGA concentration. In addition, all tested concentrations of Poloxamer 407 resulted in paste formation with liquid-like behavior after deposition, making it difficult to maintain structure or pattern [90]. In another study, compression modulus was found to improve with the use of polylactide (PLA) microcarriers in gelatin methacrylamide-gellan gum MSC-laden inks (Fig. 8D). In addition, high cell concentration and viability as well as osteogenic differentiation with matrix deposition were observed using the composite ink [91].

##### 2.1.4.1 Silicates

Silicates have been incorporated in some biomaterials to impart shear-thinning, self-healing, and capability to tune mechanical properties of biomaterials during and after printing. For example, clay nanosheets (30 ​nm in diameter and 1 ​nm in thickness) can greatly enhance strength, elasticity, toughness, and flow properties of Kappa-CA and GelMA inks through ionic-covalent entanglement strengthening mechanism (Fig. 8). Multicomponent inks comprising silicates can not only be used to print structures with better mechanical properties (Fig. 8(B)) but also to maintain high cell viability of encapsulated cells over the course of 4 months [92]. In another example, clay nanosheets were used to crosslink the polymer chains of poly(*N*-isopropyl acrylamide) (PNIPA), and the resulting hybrid hydrogel was able to stretch to up to 1424% of its original length. In comparison, the original polymer hydrogel was weak and brittle [93]. Other silicates such as polyP.Ca^2+^-complex and orthosilicate (silica) or biosilica were also used in alginate/gelatin hydrogel and bioprinted with BaG and SaOS-2 cells [94]. The results suggested that the BaG increased cell proliferation and mineralization in bioprinted SaOS-2 in hydrogels. On the other hand, no change in cell growth was noticed when no BaG but only silica, biosilica, or polyP.Ca2^+^-complex was added to alginate/gelatin hydrogel encapsulating SaOS-2 cells.

**Fig. 9:**
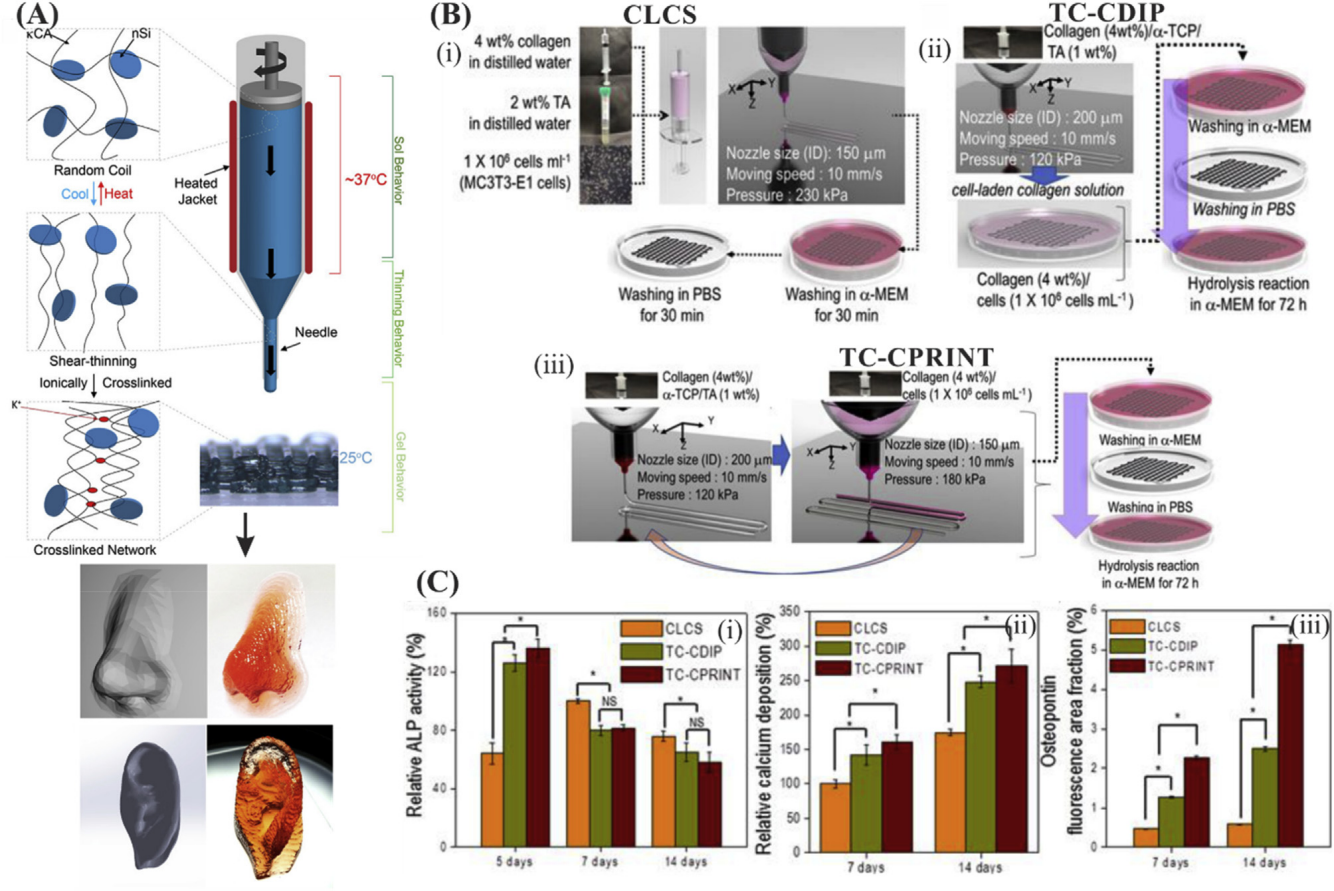
(A) Schematic illustration of reversible gelation of the shear-thinning bioinks consisting of κCA and nSi. Double helical structure was formed with controlling temperature and the structure can ionically crosslink through K^+^ ions. This allowed the 3D printing of highly complex structures. Reprinted from Wilson et al. [95] with permission from the American Chemical Society. (B) Representation of the fabrication scheme of (i) cell-laden collagen scaffold (CLCS), (ii) cell-laden α-TCP/collagen fabricated through cell dipping process (TC-CDIP), and (iii) cell-laden α-TCP/collagen fabricated through cell printing process (TC-CPRINT). (C) Comparing the osteogenic activity of collagen vs. α-TCP/collagen scaffolds in terms of (i) ALP activity, (ii) relative calcium deposition, and (iii) osteopontin (OPN). Reproduced from Kim et al. [105]. ALP, alkaline phosphatase.

Silicates, such as lithium sodium magnesium silicate, can be used for modifying rheological properties of inks. New developments in this research area include the introduction of a new family of shear-thinning hydrogels that have also reversible thermal properties (Fig. 9A) that were developed from κCA and nanosilicates (nSi or Laponite XLS) [95]. The latter silicates conferred shear-thinning properties to hydrogels such as those observed with GelMA [96]. It was found that human MSC differentiation to bone lineage can be induced by the use of nanosilicate particles in bioprinted GelMA even 21 days after bioprinting. It was observed that the degree of mineralization was dependent on the concentration of silicate nanoparticles, with best results were obtained with the use of 100 ​mg/mL silicate nanoparticles [11]. Na+, Mg^2+^, Si(OH)_4_, and Li resulting from synthetic silicate dissociation in aqueous media may also induce osteogenic cellular responses [97]. For example, the advantage of silicon oxide and magnesium oxide release from 3D printed TCP scaffolds was demonstrated as accelerated bone formation and increased angiogenesis in implants in rat models [97], [98].

##### 2.1.4.2 Hydroxyapatite

HAp is a calcium apatite and a major component of the native osseous tissue. Hence, it has widely been used in bone 3D printing [99]. The use of HAp was associated with more osteogenesis, when either BaG or HAp were used in bioprinted human MSC-laden PEGDMA [88]. HAp was also incorporated into alginate to form porous structures stimulating chondrocytes and thereby secreting calcified matrix. The latter study verified printability of the composite bioinks [100]. Superior printability was also reported with the use of GelMA/HAp and HA/HAp bioinks through microextrusion 3D printing. The structures were found to perform well in bone matrix remodeling, which makes it a good candidate for bone 3D printing [101]. Mixing HAp with gelatin bioinks was also demonstrated to enable the fabrication of 3D scaffolds with homogeneous mineralization as well as high cell viability [102]. As another example, scaffolds made of alginate/gelatin/HAp bioink were used for repairing large bone tissue defects and showed significantly improved osteogenic differentiation of stem cells [102].

##### 2.1.4.3 Tricalcium phosphate

TCP is of significant interest for use in implants and bone tissue constructs thanks to its ontogenesis induction properties and biodegradability [103]. Besides osteoconductivity, α-TCP is characterized by higher solubility than β-TCP [104]. When exposed to aqueous medium under neutral pH, α-TCP resulted in the formation of calcium-deficient HAp [49]. Thus, it was used in 3D bioprinting of bone tissue constructs. For instance, extruding deposition of α-TCP paste was used to develop a construct core that had a bioprinted shell composed of preosteoblast (MC3T3-E1) cell-laden alginate hydrogel [49]. Preosteoblast (MC3T3-E1) osteogenic differentiation (as indicated by alkaline phosphatase [ALP] activity, osteopontin, and calcium deposition) was higher for cell-laden α-TCP-collagen scaffolds as compared with bare collagen scaffolds (Fig. 9B and C) [105].

##### 2.1.4.4 Bioactive glass

BaG has been a traditional material for reconstructing bone defects [106], [107]. BaG has been proved to differentiate various cells, such as MSCs and dental pulp cells into osteogenic lineage cells and promote bone regeneration [107], [108], [109], [110], [111], [112], [113]. Various bioinks have been developed by mixing BaG with other polymers to utilize its osteogenic ability. Midha et al. prepared 3D printed bone constructs using bioinks containing silk fibroin and BaG and demonstrated superior osteogenic differentiation ability of MSCs [114]. Wang et al. showed similar results toward ​SaOS-2 ​cells, where BaG led to increased proliferation and mineralization of bioprinted cells. However, in other studies, 3D-bioprinted BaG nanoparticles mixed with PEGDMA gels was associated with lower cell viability (63.80 ​± ​7.54%) as compared with HAp nanoparticles (cell viability of 86.62 ​± ​6.02%), which is possibly because of higher cytotoxicity of BaG [88]. In another study, HAp was also demonstrated to be associated with better cell viability, proliferation, and osteogenic differentiation (mineralization after 21 days in culture) than BaG [47], [94]. Further studies should be performed to confirm effects of BaG on cell viability, differentiation, and function.

##### 2.1.4.5 Carbon nanomaterials

**Fig. 10:**
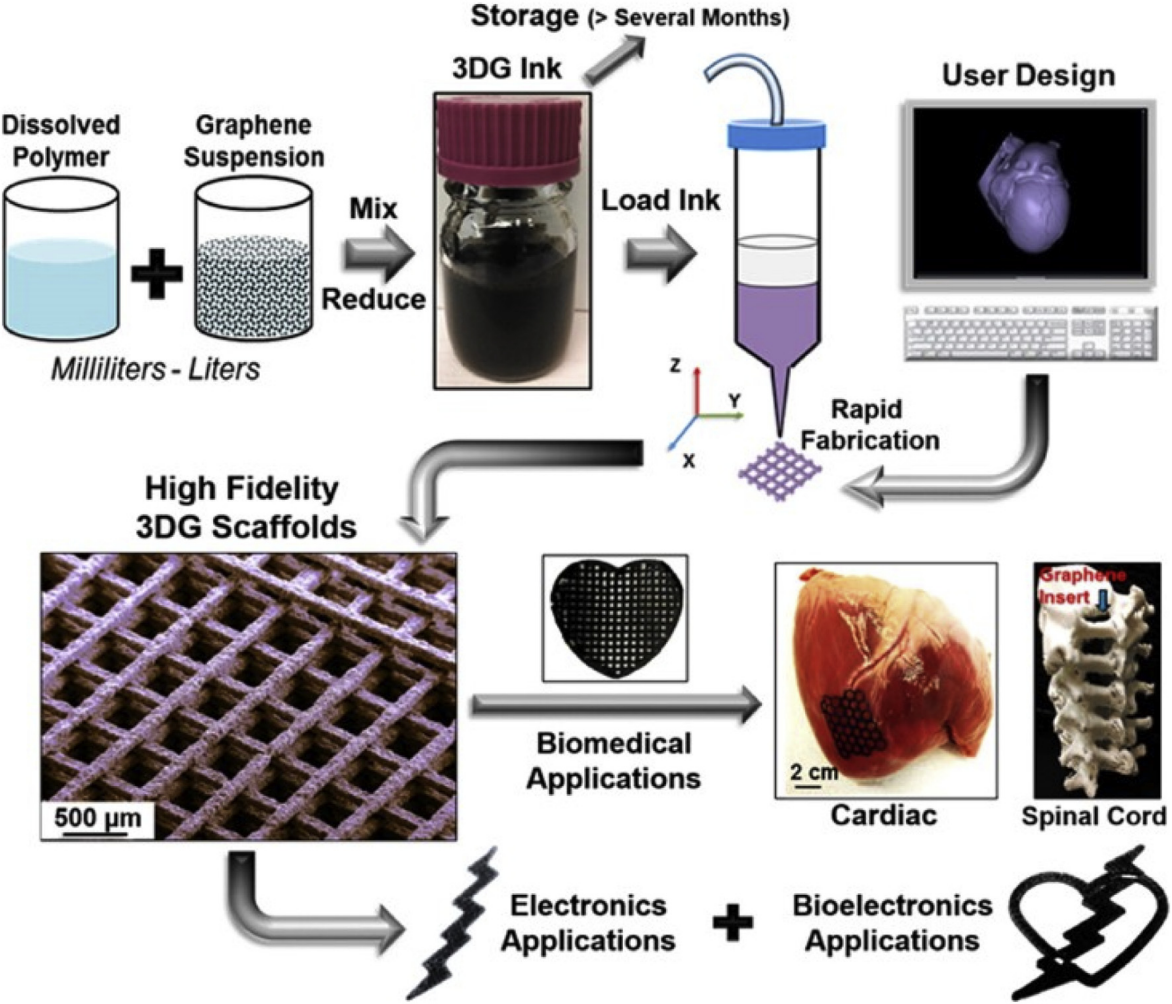
Scalable process of ink development by combining elastomer solution with graphene for fabricating porous and conductive scaffolds for tissue engineering applications. Reprinted from Jakus et al. [117] with permission from the American Chemical Society.

Carbon-based nanomaterials have been frequently incorporated into the inks particularly for neural and muscle tissue engineering applications because of their excellent electrical and mechanical properties (Fig. 10). They can also be used to control ink viscosity and thereby improve printability. Low content of graphene-incorporated into polyurethane (PU)-based hydrogels was found to result in significant (2–4 fold) increase in oxygen metabolism as well as neural differentiation of neural stem cells [115]. A study on 3D scaffolds made of PU and graphene oxide (GO) demonstrated that the GO can stimulate spontaneous myogenic differentiation [116]. Reports on highly concentrated graphene-PLGA scaffolds also allowed printing features down to 100 ​μm and exhibited significant upregulation of neural and glial genes along with superior human MSC adhesion and proliferation [117]. In another study, enhanced neural differentiation through a well-defined architecture of 3D bioprinted GelMA/graphene bioinks was reported [118]. The results suggested that these biomaterials can be further expanded to application in developing smart nerve guidance by using hybrid graphene constructs that taking advantage of multiresponsive four-dimensional (4D) bioprinting [119].

Apart from neural tissue regeneration, GO embedded into a GelMA/PEGDA matrix was reported to promote chondrogenic differentiation of MSCs in the scaffolds that were fabricated using stereolithography-based bioprinters [120]. The cell attachment was improved using printed scaffolds that were made of graphene/poly(ε-caprolactone) (PCL) composites and chemically treated with NaOH (to augment hydrophilicity) [121]. Other composite materials, such as graphene/PCL can also serve as a potential substrate for 3D printing that can be achieved through a single-step ring-opening polymerization of caprolactone in the presence of GO [122]. They were identified to be suitable for bone regeneration as the graphene/PCL scaffolds can promote osteogenic differentiation [123]. GO nanoparticles were also introduced as vehicles for delivering small drug molecules, and thereby, they can be used for protecting cartilage in 3D printing constructs. For instance, bioprinted GO nanoparticle-containing hydrogels were implanted into the knees of rats and GO nanoparticle as a BMP-7 carrier showed prolonged release of BMP-7 [124].

#### 2.1.5 Bioinks for 4D printing

4D bioprinting enables producing tissue constructs that can change in either shape or functionality over time with or without external stimulus. Bioinks comprising of smart/responsive materials with self-folding or self-assembly characteristics are potential candidates for use in 4D bioprinting [125]. Drug delivery devices as well as tissue regeneration have particularly benefited from 4D printing [126], [127]. In addition, more complex characteristics of the native tissues can be mimicked by adding the ‘time’ dimension. A significant amount of research in the literature focuses on materials deforming in response to external stimulus [128]. These stimulus include changes in temperature [129], electric and magnetic field [130], and humidity [131].

One of the major applications of 4D printing in tissue biofabrication is making blood vessels. This basically relies on advances made in self-folding polymers along with the self-organizing characteristics of cells. For instance, cell-laden hydrogels can be printed into a planar shape, which then turns to a cylindrical shape upon exposing the external stimulus. Cell-laden cylindrical hydrogels can also be printed and lead to mature vascular systems upon their activation by soluble factors [132]. The other source of time-dependent deformation can come from encapsulated cells. For instance, cell traction forces have been shown to be significant enough to induce folding of 3D cell-laden microstructures [133]. In the latter study, bovine carotid artery endothelial cells along with human umbilical vein endothelial cell (HUVEC) were used to fabricate cylindrical tubes mimicking vascular tissues.

The precise control of drug elution can be facilitated using the concept of 4D printing thanks to the folding and unfolding capabilities of stimulus-responsive materials. One of the examples include ‘multisomes’, which are basically aqueous droplets encapsulated within small oils [134]. Printing such multisomes in water makes the aqueous droplets to adhere to each other forming interface bilayers where the encapsulated components within the droplet releases upon change in pH or temperature.

### 2.2 Multicellular and stem cell–based bioinks

The selection of appropriate cells for 3D bioprinting is crucial for ensuring the success any fabricated construct. Because the native tissues are normally composed of different types of cells, the production of a biomimetic construct should involve the use of different cells. In bioprinting, cells can be used as individually encapsulated, as cells in scaffolds or as cell aggregates (spheroids) [9]. The application of droplet-based devices that enables the encapsulation, incubation, and manipulation of single cells in pico to nanoliter drops [135] for bioprinting [136] is a recently developed method, which would open new approaches to make organs/tissues in a block by block manner (modular tissue engineering) in single cell resolution.

High-resolution and droplet-based systems for cell encapsulation have recently attracted much attention. For example, Villar et al. presented a system that consisted of a static piezoelectric actuated droplet generator, which ejected droplets from an oil-immersed nozzle into a lipid-in-oil bath [137]. Using their system, human embryonic kidney and ovine MSCs were printed reaching a high droplet resolution of 1 ​nL. Although the authors achieved high resolution of droplets, the system could be improved by combining extrusion-based systems. The high-resolution capability of droplets for 3D bioprinting is only one of the requirements for the development and production of biomimetic and complex tissue structures. Various techniques of multicomponent bioinks combined with droplets systems would have an impact on tissue engineering approaches for complex tissue biofabrication.

Spheroid bioinks can be assembled into larger structures using assembly or fusing procedures. Yu et al. [138] demonstrated a novel tissue spheroid bioink produced by a self-assembly process without using any harsh chemicals as crosslinker or support materials. The spheroids were able to form tissue strands up to 8 ​cm long with rapid fusion of the cells. The techniques prior to 3D bioprinting face great challenges of making tissues in large scale or spatial organization of cells. The cell encapsulation accompanied by bioprinting techniques, besides giving its scalability and reproducibility, also allows the possibility of accurately selecting the spatial location of specific cells in scaffolds. One of the barriers in increasing the scale of cell-laden scaffolds is the lack of vascularization, limiting the nutrients needed for the tissue maintenance. Successful fabrication of a vascularized tissue construct requires synergy between high throughput, high-resolution bioprinting of larger perfusable channels and instructive bioink that promotes angiogenic sprouting and neovascularization [139], which requires the presence of diverse cell lines in strategic regions of the tissue.

Although different types of cells can be used in bioinks, the use of stem cells offers different advantages as they can be obtained from various sources and differentiated into various lineages. Although BMSCs have widely been used for tissue engineering, there are other potential sources of MSCs, such as tissues, which are usually discarded, such as fat tissue after liposuction, cord blood, or after inferior turbinate removal for treatment of nasal obstruction [140]. For example, hTMSCs were used in bioprinting [58] and had high yield (30 ​+ ​1.2-fold increase in nasal septal progenitors) relative to BMSCs [141] with multilineage differentiation potential [140]. In addition, donor age and passage had no significant effect on their differentiation characteristics (unlike BMSCs or adipose tissue-derived MSCs [aMSCs]) [142]. Lim et al. evaluated the use of hTMSCs in experimental acute ischemic stroke [143]. According to their conclusions, hTMSCs could improve functional recovery following ischemic stroke. In previous work, Lim et al. also demonstrated the hTMSCs ability for cell survival and osteogenic differentiation when placed in 3D printed structures [144]. These findings make hTMSCs attractive source for use in regenerative therapeutics and 3D bioprinting. Although multipotent stem cells are the most commonly used in tissue engineering, pluripotent stem cells also offer wider potential. This is especially true after the development of induced pluripotent stem cells (iPSCs), which help to avoid several problems that are classically associated with the use of embryonic stem cells. Moreover, iPSCs were used to derive MSCs, and this represents an attractive source for MSCs because it can circumvent the problem of limited initial number of autologous MSCs obtainable from classical sources, such as bone marrow. Interestingly, iPSC-derived MSCs are also rejuvenated during the process of reprogramming leading to better survival, proliferation, and differentiation capabilities [142]. These progresses in stem cell technologies can potentially provide cell source alternatives for use in 3D bioprinting for personalized medicine.

One major issue that should be taken into consideration when implanting bioconstructs *in vivo* is their vascularization for better cell survival and function. Engineering vascular network into bioprinted constructs represents a viable solution. Different types of cells, such as HUVECs, human neonatal dermal fibroblasts, and 10T1/2 fibroblast cells, were used with GelMA to develop vascularized constructs [19]. Constructs including varying types of cells to develop more biomimetic constructs were developed.

#### 2.2.1 Dynamic hydrogels for multicellular 3D bioprinting

Under the native microenvironment, the spatial distribution of cells determines the communication between cells, which affects cell function, growth, and differentiation. For 3D bioprinting, it is important to control the spatial distribution of different cell types in defined locations to be able to mimic cell arrangement in the native tissues. Tekin et al. introduced a simple method to control spatial organization of multiple cell types using a thermoresponsive hydrogel [145]. They bioprinted two different types of cells, human hepatoblastoma (HepG2) cell line, and HUVECs, into PNIPA, which had a lower critical solution temperature of ∼32 ​C. Taking advantage of the shape changing properties of PNIPA at different temperatures (24 ​C and 37 ​C), the cells of the second type were spatially arranged around the cells of the first type using dynamic circular and square microwells.

### 2.3 Biomolecule-contained bioinks

In addition to bioprinting of 3D constructs that have different materials and cells, it is evident that biomolecules are needed to tune and control cell function [146], [147]. Thus, constructs having biomolecule releasing properties have been developed [148]. Hydrogels can provide the spatial and temporal control of the release of different therapeutic agents, including growth factors and drugs. Owing to the tunable physical characteristics and programmable degradability offered by hydrogels, they can be exploited as a robust platform for different physicochemical interactions with encapsulated drugs that can be used for controlling drug release [149].

Various biomolecular gradients using bioinks were successfully prepared, and they were demonstrated to be useful in directing cell differentiation and function in 3D bioprinted constructs [11]. One common strategy is to chemically or physically conjugate biomolecules such as growth factors with gradient concentrations to hydrogels. For example, Byambaa et al. prepared a bioactive GelMA bioink containing gradient vascular endothelial growth factor (VEGF) for vascularized bone tissue. They chemically conjugated VEGF with gradient concentrations to GelMA prepolymer and printed bone constructs with different VEGF distribution [11]. In another study, polystyrene microfibers were produced using a spinning process and subsequently coated with serum or fibrin and bioprinted on with BMP-2 by using inkjet bioprinter. Cells were aligned parallel to the fiber orientation. There was increased osteogenic cell differentiation of C2C12 cells compared with non-BMP bioprinted control regions [150]. Recently, Paris et al. found that biomaterial surface curvature also can be important for interface tissue engineering, such as ligament insertion to the bone [151]. Do et al. [152] used 3D printing to make a system for drug release comprising PLGA core and alginate shell in a sequential manner and showed non-toxic of the construct to BMSCs. In the following sections, the addition of different growth factors to bioinks is discussed.

#### 2.3.1 Bone morphogenetic proteins

BMPs are growth factors with multiple functionality including the development of neural, heart, and cartilage tissues as well as in postnatal bone formation [153]. For 3D bioprinting, BMPs were added into bioinks in the form of proteins or plasmids encoding BMPs. BMP-2 plasmid was combined in 3D bioprinted BMSC-laden alginate constructs [50], which was associated with osteocalcin expression. However, no bone was formed for the period of 6 weeks of implantation in the subcutis of mice although the BMP-2 protein was produced over the 7 days of culture. In another work, two-dimensionally bioprinted BMP-2 onto acellular dermal matrix (ADM) was employed to treat cranial parietal bone defects in mice. The results showed that the new bone formed on 66.5% of BMP-2 bioprinted areas of ADM when it met the cells and only on bioprinted areas with BMP-2 [154]. Similar results were also obtained with 3D bioprinted BMP-2 onto DermaMatrix™ human allograft scaffolds, where C2C12 cells were differentiated to osteogenic cells at BMP-2 areas [155].

Although BMP-2 was successfully used to enhance bone formation and it was applied clinically, one of its problems is its burst release, which is associated with quick loss of its function and the need to use larger doses with attending raised cost and complications. 3D bioprinting may help reduce dosing and possible side-effects with precisely controlled release at predetermined location. Unwanted adverse effects of excess BMP-2 may include inflammatory infiltrates and increased osteoclast-like cells resulting in the formation of cyst-like bone and soft tissue swelling [156], [157].

#### 2.3.2 Vascular endothelial growth factor

VEGF is basically an angiogenic factor that is produced by cell types such as tumor cells, platelets, and macrophages [158]. VEGF functions in bone formation [159], wound healing, as well as hematopoiesis [160]. Because a major limiting factor in the success and translation of tissue engineering into the clinic is vascularization, many strategies were developed to induce angiogenesis using VEGF. For example, a biomimetic growth factor–releasing system was proposed where electrostatically assembled recombinant human VEGF and recombinant human BMP-2 were released from gelatin by the effect of matrix metalloprotease 2 (MMP2). Upon the gelatin degradation using the MMP2, growth factors were released. It was found that ALP activity in MSCs increased more significantly, and it was sustained in the MMP-triggered BMP-2 release system for longer time, which involved crosslinked nanocoated scaffolds in MSC/endothelial cell co-culture [161].

It is important to have proper control over release of growth factors to avoid unwanted effects, e.g. high concentration of VEGF may inhibit osteogenesis [162] and excess of BMP-2 can be associated with inflammatory infiltrate and increased number of osteoclast-like cells [156]. In one example, murine neural stem cells (C17.2), collagen hydrogel, and VEGF-releasing fibrin gel were bioprinted to fabricate artificial neural tissue. C17.2 cells–embedded collagen gel was bioprinted to the VEGF-releasing fibrin gel, and morphological changes of the bioprinted C17.2 cells were examined. The bioprinted cells showed high viability (92.9 ​± ​2.3%) compared with those cells that were manually plated. Cell-containing collagen bioink was bioprinted with 1 ​mm gap from the VEGF-releasing fibrin gel, and the cells migrated toward the fibrin gel [163]. In another study, Poldervaart et al. used gelatin microparticles for controlled VEGF release. It was found that the release of VEGF from gelatin microparticles was continuous for 3 weeks during *in vitro* experiment, and bioactivity was confirmed using cell migration assays. Human endothelial progenitor cell-laden Matrigel® was bioprinted with two different regions, one region of the construct containing VEGF-loaded gelatin microparticles, and the other region did not, to serve as control. It was demonstrated that the cell migration and vascularization were distinguishable at the VEGF regions as compared with the control regions [164].

#### 2.3.3 Fibroblast growth factor

Fibroblast growth factors (FGFs) are responsible for regulating cell behavior and function, such as cell differentiation, migration, and survival. These factors are currently exploited for tissue regeneration applications and in drug delivery systems [165]. Preosteoblastic cell response to spatial patterns of FGF-2 was mediated in 3D bioprinted constructs using an inkjet printer [166]. The immobilized FGF-2 was active in biological study, and the density of seeded cells on the bioprinted patterns was related to the FGF-2 concentration. The bioprinted structures treated with the FGF were associated with promoted chondrogenic properties owing to the stimulation of cell proliferation using the FGF [167]. Another study stipulated that bioprinted FGF-2 and BMP-2 patterns can result in enhanced tenocyte and osteoblast viability of bone cells [150].

#### 2.3.4 Transforming growth factor

TGF is known to induce granulation tissue formation [168]. Gurkan et al. employed GelMA-based bioinks containing TGF-β1 and BMP-2 along with human MSCs to mimic fibrocartilage phase at the bone-tendon interface. They used nanoliter-droplet based inkjet bioprinting for fabricating a gradient of growth factors. These constructs led to differentiation of human MSCs toward osteogenic and chondrogenic phenotype in a spatial manner [169]. *In vivo* studies revealed that the addition of TGF-β to PCL/alginate gels can lead to improved ECM formation in 3D bioprinted constructs subcutaneously implanted in mice [170].

#### 2.3.5 Stromal cell–derived factor

Stromal cell derived factor-1 (SDF-1) is a member of the CXC chemokine family, which induces the migration of progenitor/stem cells and initiates the regeneration process [70], [171]. It can be also used to build *in vitro* tumor models with angiogenesis environment. Bray et al. bioengineered tumors using glycosaminoglycan-based hydrogel containing SDF-1, VEGF, and FGF-2 and several cell types. Many different types of cultured cells within this model were less sensitive to chemotherapy in contrast to 2D cultures. Tumor regression was also evident which was comparable to that observed *in vivo* [172].

#### 2.3.6 Extracellular matrix

One of the main prerequisites in 3D bioprinting is finding an appropriate bioink that provides a tissue-specific microenvironment supporting the cellular growth and maturation. The ECM is the mixture framework consisting of different components, such as collagen, glycosaminoglycans, chondroitin sulphate, and elastin [173]. Decellularized ECM (dECM) materials can be obtained from different tissues, where cells are removed by a sequential procedure leaving the ECM intact. dECM-derived hydrogels have been considered as bioinks for 3D bioprinting owing to their capability to inherit the intrinsic cues from the native ECM. Using this methodology, recently Ali et al. have reported the use of a photocrosslinkable dECM-derived bioink that can accelerate the formation of renal tissues [174]. In their method, porcine whole kidneys were decellularized through a perfusion method, dissolved in an acid solution, and chemically modified by methacrylation. The bioink formulation was developed by combining the methacrylated dECM with gelatin, hyaluronic acid, and glycerol. After crosslinking, the un-crosslinked components (gelatin, hyaluronic acid, and glycerol) were gradually washed out under the culture condition. In addition, *in vitro* results from the crosslinked kidney-based bioink showed a significant increase in cell proliferation when compared with GelMA-based bioinks. In another study, Pati et al. [175] developed dECM-based bioinks to mimic specific environments of various tissue types for tissue-specific bioink formulation. In another study, stem cell-laden dECM bioinks were developed for 3D bioprinting of prevascularized structures to improve cell interaction and thereby augment vascularization and ECM formation. The results suggested that the developed bioink can enhance cardiac repair [176]. For the dECM production, heart tissue (left ventricle) from a 6-month-old Korea domestic pig was used. Before conducting the printing experiments, the tissue was dissected, decellularized, and pH adjusted. The results demonstrated that the stem cell patch has therapeutic efficacy through improvement of cardiac function and decrease of left ventricular remodeling. According to the authors, this platform technique may open new avenues for delivering cells with high retention capability and regenerating ischemic tissue area. Although dECM bioinks provide novel opportunities to fabricate tissue specific constructs, the decellularization process requires multiple steps including precise quantification of DNA and ECM components, which increase the cost of the dECM fabrication.

#### 2.3.7 Peptide motifs

Peptide motifs are basically responsible for biomolecular interactions [177]. For example, RGD is recognized by integrins and helps endothelial cell adhesion and migration. Engineered human-safe virus nanofibers of RGD-phage was used in 3D printed ceramic (bicalcium phosphate, HAp/TCP 80/20) containing chitosan. When scaffolds were seeded with MSCs, osteogenesis and angiogenesis were induced *in vivo* [178]. RGD-phage induced MSC osteogenic differentiation without the need for any additional osteogenic media [179].

#### 2.3.8 Platelet-rich plasma

Platelet-rich plasma (PRP) consists of different growth factors including VEGF, platelet-derived growth factor, TGF, insulin-like growth factor, and SDF. All of these growth factors play important roles in inducing angiogenesis, derivation of stem cells, and tissue regeneration. In 3D printing studies, PRP was incorporated into alginate hydrogel to produce autologous/patient-specific biological factor containing constructs. Semi-crosslinked alginate/CaCl_2_ ink was printed as a 3D structure and was crosslinked in a calcium ion-containing agarose gel [180].

#### 2.3.9 Stem cell secretomes and other molecules

Stem cell secretomes are bioactive molecules released by stem cells and serve as a long-term source of important growth factors critical for tissue regeneration. These biomolecules have shown great potential in regenerative medicine therapies [181], [182], [183]. These secretomes contain various levels of cytokines, chemokines, growth factors, and angiogenic factors. The autocrine/paracrine function of these molecules plays significant role in the regulation of many physiological processes including apoptosis, scarring, and tissue revascularization as well as directing endogenous and progenitor cells to sites of injury [184]. There has been a research trend toward ​the development of secretome-based therapeutical strategies to repair or restore salivary glands (SG) damaged by radiotherapy. For this purpose, novel 3D bioprinting approaches have been developed to assemble the SG cells in co-culture and produce 3D tissue compartments and ductal structures that resemble mini-SG [185]. Furthermore, 3D bioprinting systems have been employed in cultures with oral stem cells, such as human dental pulp stem cells in combination with secretome components, including FGF-10 to enhance α-amylase-secreting cells [185].

Integration of bioinks with bioactive molecules is a good choice for use as bioink for stem cell bioprinting. For instance, gold nanoparticles with gelatin and thiolated HA were employed with fibroblasts to bioprint vascular structures [186]. In another research by Mannoor et al. silver nanoparticles used for bioprinting a 3D bionic ear [187].

## 3 Methods to fabricate heterogeneous constructs

**Fig. 11:**
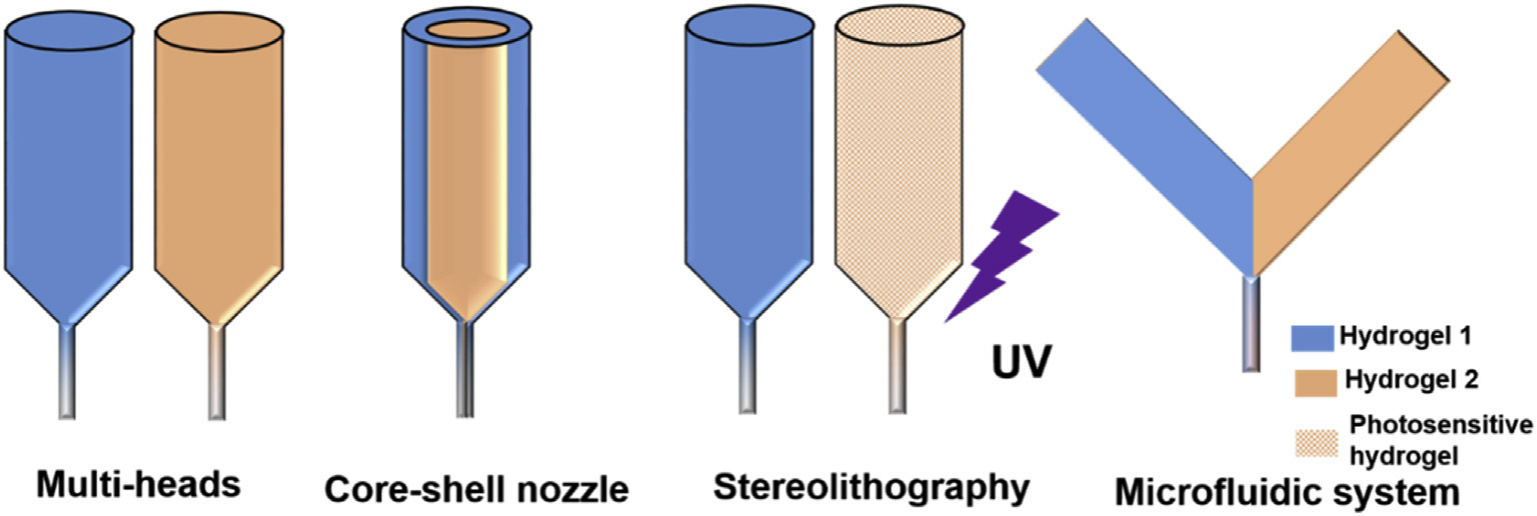
Schematic illustration of different 3D printing systems that have been used to produce multimaterial constructs.

To develop successful biomimetic and heterogeneous constructs, it is necessary to have appropriate fabrication tools and methods. The principle and application of each technique together with their advantages/disadvantages are introduced below. Early attempts to develop multimaterial constructs relied on the use of sequential printing or on the use of multiple printing heads (Fig. 11). However, these methods have limited capabilities as they lack high resolution, physical/chemical integration at the interface between materials, mechanical stability during the printing process and/or crosslinking, and gradient material properties.

### 3.1 Multihead systems

**Fig. 12:**
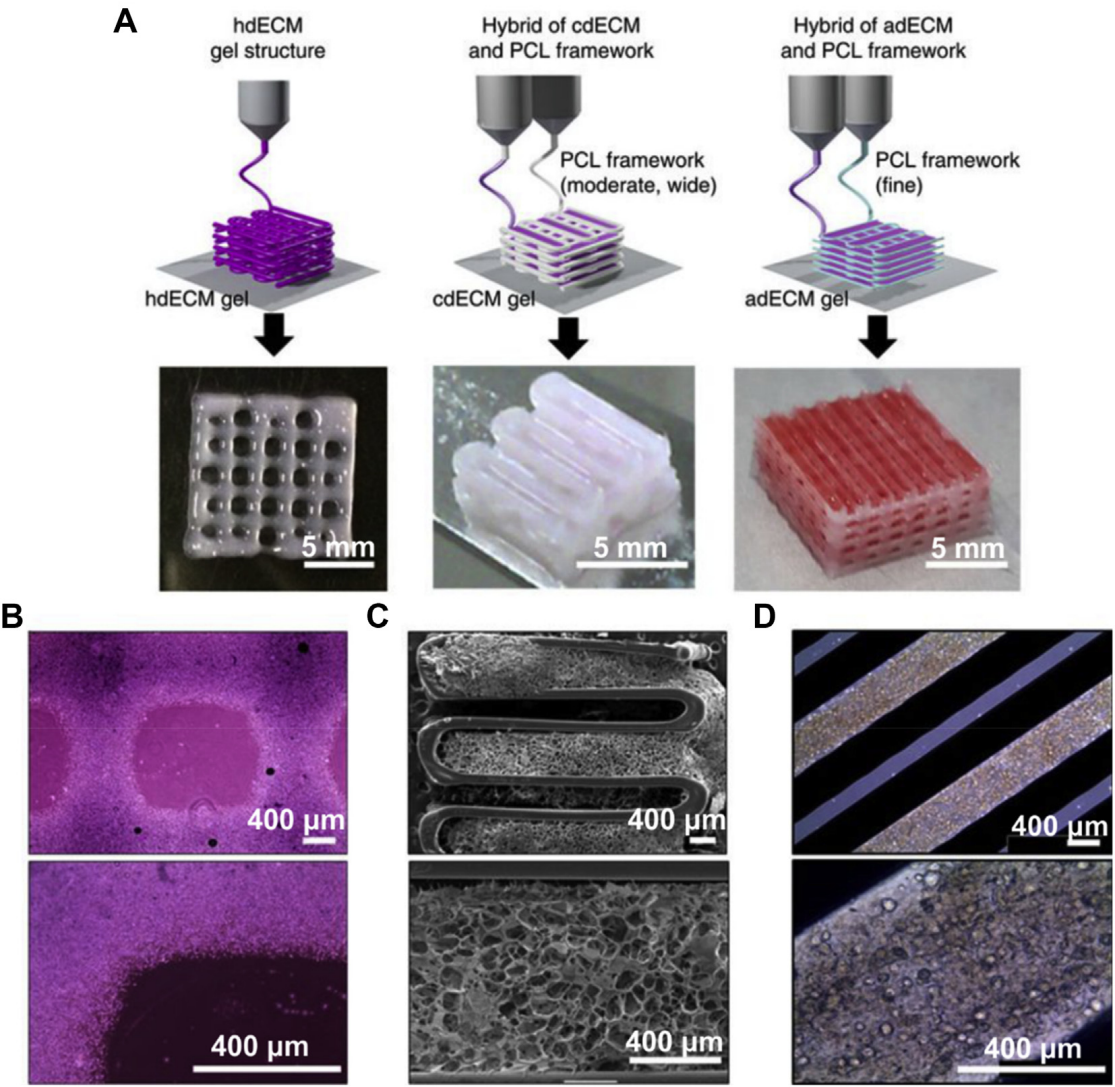
Three-dimensional printing with (A) single and (B, C, and D) multiprint head systems [175]. PCL, poly(ε-caprolactone); dECM, decellularized ECM

With the development of 3D printers, it is now possible to develop multihead systems for fabricating heterogeneous structures with several bioinks. The construction of multimaterial architectures often involves sequential printing of individual materials using multiple nozzles. Cells are usually premixed with hydrogels, and different bioinks can be bioprinted together. Pati et al. printed cell-laden constructs with dECM-based bioink and PCL using a multihead system (Fig. 12) [175]. The PCL was bioprinted as a framework and cell-laden dECM was placed between the PCL layers. By providing an optimized microenvironment, specific tissues such as adipose, cartilage, and cardiac tissues with high cell viability and functionality can be formed.

The main drawback of this method is that only one material can be printed at a time. This results in having a relatively slow printing process, which limits the use of this method in the fabrication of multimaterial constructs. In addition, the change of different heads requires careful alignment of nozzles and start-and-stop ink flow with no defects introduced. These issues represent major limitations of multihead printers in bioprinting heterogeneous constructs [18], [188].

### 3.2 Core-shell needle system

3D printing utilizing coaxial needles has been used to fabricate structures with core-shell, heterogeneous, and hollow strands [189], [190]. With core-shell bioprinting, the mechanical properties of pure materials can be greatly turned. For example, core-shell PEGDA/alginate hybrid inks (PEGDA as the shell and alginate as the core) showed higher strengths and tensile moduli compared with alginate. The printed constructs by the core-shell bioinks led to shape-recovery upon removal of applied deformation. In addition, an ECM-like hydrogel with cells could be printed as the core while the shell would protect the cell-laden hydrogel and retain the structural integrity of the cell-incorporating hydrogel [189]. A core-shell bioprinting platform was also used to generate MSC nanospheres through a GelMA/PEGDA bioink system. TGF-β1 was included in the nanospheres by using electrospraying technique. The study claimed that PEGDA can lead to better printability and increased compressive modulus of the 3D bioprinted constructs [191]. Other examples of core-shell constructs include the production of alginate/nHAp structures. The latter approach of core-shell bioprinting offered support to protein release and, hence improved cell attachment/growth [192]. Moreover, core-shell fabrication technology can be useful to make tissue-like constructs for drug studies. For instance, glioma stem cell-laden alginate was fabricated using this technology for drug resistance studies [193].

### 3.3 Stereolithography

Stereolithography (SLA) is a 3D printing technique that employs UV light to cure photosensitive polymer resins. SLA enables the creation of defined geometries with high resolution. It should be mentioned that rheological characteristic required for SLA method is different from that required for extrusion bioprinting. This needs to be considered when developing corresponding bioinks. Yeong et al. employed SLA to bioprint tumor cells and hepatocyte spheroids, and they found that it was possible to achieve high cell viability at 72 ​h for both cell types. Furthermore, liver-specific functions of hepatocytes were also maintained [194]. A new SLA approach that enabled high resolution multimaterial 3D printing using projection microstereolithography (PμSL)-based additive manufacturing system has recently been developed [194]. In this technique, the PμSL system with high lateral resolution of up to ∼1 ​μm was used to generate and reconfigure light patterns using a digital microdisplay (DMD) device as a dynamic photomask. The system was able to convert liquid monomer resin into solid in a layer-by-layer fashion.

Multimaterial printing was also possible via material exchanging system integrated into PμSL system. Ge et al. demonstrated multimaterial 4D printing using shape memory polymer and high resolution PμSL. Photocuring methacrylate-based shape memory materials were printed with automatic material exchange system resulting programmed shape shifting architectures [195]. Recently, Miri et al. reported on a bioprinting platform for fabrication of heterogeneous constructs with different cell-loaded hydrogel bioinks using DMD and PμSL [196]. They used microfluidic device for switching between multiple bioinks containing different cells and hydrogels to achieve layer-by-layer multimaterial bioprinting. Various cell types including osteoblasts, fibroblasts, MSCs, MCF7, and C2C12 were embedded in GelMA bioinks having varying concentrations, and they were bioprinted into 3D heterogeneous constructs [190], [196]. It was demonstrated that this printer is capable of bioprinting constructs by using 2–3 bioinks in 20 ​s, which is faster than industrial or manually operated bioprinters, which take minutes to do this [197]. The system may help to develop vascularized structures such as vascularized tumor models or tendon-bone interface using three cell types (MSCs, fibroblasts, and osteoblasts).

### 3.4 Digital light projector

Digital light processing (DLP) method offers high speed and resolution biofabrication scheme, which is favorable when scalable production is needed [198]. In terms of working mechanism, it is basically identical to SLA, though it uses a visible light source for photopolymerization [199]. DLP-based bioprinting has thoroughly been addressed by Zhu et al. [200]. In their study, a high speed biofabrication of prevascularized tissue constructs comprising of vascular channels was shown allowing highly complex features and controllable distribution of multiple cell types (endothelial cells and MSCs). Moreover, the fabrication of vascular graft models was demonstrated by using cell-laden hydrogel based on photocrosslinkable poly(ethylene glycol-co-depsipeptide) (PEG-co-PDP). In this approach, the physical properties of the hydrogel were controlled simply by the exposure time [201]. Adding 1 w/v% silk fibroin particles was found to increase the viscosity of the GelMA solution by two folds allowing to keep the cells retarded within the hydrogel for DLP printing [202]. The hydrogel showed significant level of metabolic activity and non-cytotoxicity was confirmed by the biocompatibility studies.

Further studies have addressed visible light photocrosslinking of methacrylated poly(vinyl alcohol) and GelMA [203]. Cell-laden hydrogels were printed with a fine resolution while complex features were retained. The use of encapsulated stem cells proved the potential applications in bone and cartilage tissue engineering. Moreover, allylated gelatin was shown to have capabilities to serve as a thiol-ene clickable chondrocyte-encapsulating bioink [204]. The dimerization network allowed the use of visible light initiation system for hydrogel formation leading to high shape fidelity.

### 3.5 Multimaterial microfluidic bioprinting

Microfluidics technology can control flow of different bioinks integrating multimaterials into fibers or droplets that contain different cell types or ECM components. Combination of a microfluidic printhead with a 3D printing system has been reported by employing the viscoelastic PDMS ink contrary to the cell-laden bioink [205]. The relation between printing parameters (namely printing speed and applied pressure), bioink rheology, and filament composition can be determined by mathematical models [205]. Colosi et al. further developed this method and introduced low viscosity bioinks into the microfluidic system. Using this system, heterogeneous constructs can be bioprinted, and desired bioink type can be selected and sent to the extruder using coaxial nozzle. Furthermore, multimaterial extrusion bioprinting platforms were developed, which can be used for bioprinting of up to seven different types of bioinks that can switch fast and smoothly with various reservoirs and rapid fabrication of complex tissue-like structures [18]. Fabrication of multicomponent structures (15 times faster than conventional nozzle-based modalities) was achieved by integrating a digitally tunable pneumatic single-print head with the bioprinting system.

In terms of printing speed, microfluidic bioprinting is one of the fastest approaches (Table 1). Moreover, shear-thinning and chemical gelation are the most common gelation mechanism methods in multihead bioprinters and microfluidic systems [18]. Theoretically, there is no limit for the maximum number of cell types or biomaterials that can be bioprinted with the use of multi-head systems. However, a limited number of cells or materials have been printed using multihead-based system because of limited time for printing process [18]. In particular, the use of up to seven bioinks continuously with fast and smooth switching material between different reservoirs was reported for microfluidic bioprinting [18], [205].

**Table 1:** Comparison of different multimaterial bioprinting systems.

Multibioinks bioprinting system	Multiheads system	Stereolithography	Microfluidic bioprinting
Printing speed	Low	Low to medium	Fast
Gelation methods	Chemical, shear-thinning	Photocrosslinking	Chemical, shear-thinning
Maximum cell types based on current reports	3 types of cells	2 types of cells	–
Maximum material types based on current reports	3 types of materials	2 types of materials	7 types of materials

### 3.6 Sacrificial template assisted printing

Apart from direct printing methods, other hybrid approaches have been shown in previous studies to shape biomimetic tissue constructs. For example, carbohydrate glass was shown to provide supportless printing capability [206] and used as a cytocompatible sacrificial template network to form cell-laden hydrogels representing vascular channels. Endothelial cells encapsulated in different hydrogels, such as alginate, have led to successful formation of 3D vascularized tissue constructs when using carbohydrate glass templates [207], [208].

## 4 Tissue fabrication using multicomponent bioinks and technologies

In this section, we describe some important applications of multicomponent bioinks and related technologies to fabricate tissue constructs. We are not going to cover all tissues and organs as it is beyond the scope of this review. Interested readers are referred to other review papers to know more details about applications of 3D bioprinting materials and technologies in tissue fabrication [45], [209], [210], [211].

### 4.1 Heart

Heart is a vital organ in the body and is comprised of multiple cells including fibroblasts, endothelial cells, cardiomyocytes, smooth muscle cells, and pacemaker cells structurally organized in a mixture of ECM materials [212]. 3D bioprinted heart tissues using multicomponent bioinks have been tested *in vitro* and *in vivo* and showed vascularization and functional preservation. For instance, Gaetani et al. reported 3D bioprinted hyaluronic acid and gelatin patches containing cardiac progenitor cells, and they implanted those patches into mice hearts [213]. The *in vivo* results showed good cell survival/engraftment and increased cardiac and vascular differentiation markers after 4 weeks. The MRI and histology studies indicated improvements in cardiac function after implanted of the cardiac patch. In another example, Gaebel et al. bioprinted a cardiac patch using two types of human cells (HUVECs and MSCs) and reported better cell viability and increased vessel formation [214]. They implanted the patch in the infarcted zone of rat hearts and primitive vascular networks were observed in 3D bioprinted myocardium after 8 weeks. More importantly, the cardiac patch enhanced the angiogenesis in the border zone of infarction and preserved cardiac function after acute myocardial infarction. iPSC-derived cells have also been used to fabricated cardiac patches using 3D bioprinting. For instance, Gao et al. fabricated a human cardiac muscle patch with iPSC-derived cardiomyocytes, smooth muscle cells and endothelial cells using 3D pronting and found calcium transients and beating synchronously were generated after one day [215]. After implantation of the patch into the heart of mice with surgically induced myocardial infarction, cell engraftment rates in the patch was 11% after 4 weeks, and the cardiac function and vascular and arteriole density were much higher than cell-free scaffold group. In another study, cardiac patch bioprinted with iPSC-derived cardiomyocytes, fibroblasts, and endothelial cells showed similar results [216]. After implantation into mice heart, integration, and vascularization were observed.

### 4.2 Liver

Liver is a key organ in the body's metabolism (performed mainly by hepatic parenchymal cells) and has a good capacity for self-regeneration. Though, when it comes to severe injury and chronic damage, it may fail to regenerate properly and requires liver transplantation. Currently, there is a pressing need for developing the liver structures that can ensure effective drug metabolism function and can allow monitoring hepatotoxicity and metabolite production *in vitro* [217]. To this end, Ma et al. developed a microscale hepatic construct by using 3D bioprinting of various cell types in a predefined biomimetic manner. Bioinks containing human iPSC-derived hepatic progenitor cells with HUVECs and aMSCs in hydrogel were bioprinted to microscale hexagonal units, which consisted of liver cells and supporting cells [218]. Another study addressed the use of multihead bioprinting system for liver tissue engineering by integrating a 3D cell-laden construct involving PCL as a mechanically efficient substrate for cells (i.e. ​hepatocytes, HUVECs, and human lung fibroblasts) [219]. The PCL was used for improving the mechanical integrity of the bioprinted constructs. The results suggested that there was a great potential of the designed bioink for inducing heterotypic cellular interaction within the 3D bioprinted construct.

### 4.3 Cartilage and osteochondral tissue

Many attempts have been made to mimic the complex structure of the native cartilage by using 3D bioprinting. Cartilage tissue functions as an interface in joints to reduce friction and it acts as a damping material. Owing to the lack of vascularization in the native articular hyaline cartilage, its regeneration is limited, and hence, there is a need to develop novel methods for treatment of articular cartilage defects [220]. For this purpose, HA has been widely used as hydrogel for cartilage regeneration. Shie et al. printed HA with PU and cured the 3D constructs with light [221]. The scaffold was found to have high cytocompatibility because of differentiating MSCs into chondrocyte and also closely mimicking the mechanical properties of articular cartilages. For cartilage tissue, PCL was also used to improve mechanical properties of chondrocyte-laden hydrogels. Bioinks containing PCL and chondrocyte-laden alginate were bioprinted, and the cells were maintained well in the scaffold. In addition, it was noted that PCL-alginate gels containing TGF was found to be accompanied by higher ECM formation [170]. In another study, chondrocyte-laden fibrin-collagen hydrogel was 3D bioprinted on electrospun PCL nanofibers. It was found that fabricated constructs were associated with the formation of cartilage-like tissue both *in vitro* and *in vivo* [222].

Reconstructing osteochondral defects is a major challenge in cartilage tissue engineering. It is expected that multimaterial and multicellular bioprinting would have a major impact on treating such defects by providing 3D osteochondral constructs that can have appropriate and biomimetic multicomponent structure and composition with improved success and durability potential. Reconstruction of meniscus and its insertion to the bone can also be another important application of 3D printing in cartilage tissue engineering. In this regard, low cost 3D printers have been shown to be applicable for printing patient-specific cartilage tissue constructs (made of poly(2-acrylamido-2-methylpropanesulfonate) and polyacrylamide) [223]. Levato et al. used GelMA-based hydrogels for 3D bioprinting of scaffolds for cartilage regeneration. They cultured articular cartilage-resident chondroprogenitor cells, BMSCs, and chondrocytes. They demonstrated that it is possible to 3D bioprint constructs having a distribution of collagens and glycosaminoglycans using such co-culture system [91]. Multicellular structures with chondrocytes and osteoblasts also need different ECM components for each cell type. Thus, HA hydrogel was used for the chondrocytes, and collagen type I hydrogel was used for the osteoblasts. Cell viability of each type of cells in proper hydrogel environment was more than 90% after 14 days *in vitro* [91].

### 4.4 Bone tissue

Bone tissue is comprised of multiple elements that play different roles, such as periosteum, osteons, and medullary cavity [224]. Fabricating such a complex material that can both support cells and act as a load bearing structure requires the utilization of multiple ingredients in 3D bioprinting process. A large variety of ceramic, metallic, polymeric, and composite materials were thus employed for 3D bioprinting of implants that were investigated in bone repair [14], [98], [225], [226], [227]. For example, a combination of hydrogels, in the form of alginate-gelatin ​and HAp was loaded with MSCs, and the resulting bioink was used to successfully 3D bioprint stable constructs. It was found that the cell viability remained high after 3 days in culture [228].

Mimicking bone muscle/tendon interface has been a major challenge because of the sophisticated and heterogeneous tissue architecture of the interface tissue. Conventional methods for treating tendon injuries include primarily suturing. However, this method suffers from low adhesion and complicated inflammation [229], [230]. Reproducing such structure requires a full control on biomaterial and cell gradient. In one example [231], muscle/tendon replacement was 3D printed using a multimaterial platform. Thermoplastic PU (mimicking muscle) and PCL (mimicking tendon) sides were head-to-head incorporated in the printed scaffold. The fabricated replacement demonstrated high cell viability against C2C12 cells and NIH/3T3 cells with mechanically graded stiffness matching the native muscle tendon structure. Another study addressed bone/tendon interface reconstruction by an interdigitated patterning of microdroplets [169]. The structures consisted of two bioinks based on MSCs combined with BMP-2 and TGF- β1 on a hydrogel substrate were made. It was found that a controllable biochemical gradient that mimics the native fibrocartilage was obtained. Controlled co-bioprinting of tenocytes and myoblasts for engineering of muscle and tendon tissues has been also demonstrated [232]. In summary, the cell suspensions were bioprinted on prebioprinted bioinks of pure GelMA and GelMA-PEGDMA. The results revealed that the myoblasts were well connected to the tenocytes except those at the sample boundaries because of the weak interface caused by the tensions developed in the muscles without having a supporting ECM.

### 4.5 Adipose tissue

Engineered adipose tissue constructs can be used in plastic and reconstructive surgery for the reconstruction of soft tissue defects or for aesthetic indications. Adipose tissue has many roles in the body among which a support of the surrounding tissues and organs is an important one [233]. In addition, it functions as a means for energy storage and metabolic functions. Bioinks containing decellularized adipose tissue and MSCs have been 3D bioprinted to produce precisely defined and flexible dome-shaped constructs, which were found to have high cell viability over the period of 2 weeks. Moreover, adipose tissue was formed after their implantation in mice [234]. Narayanan et al. developed a bioink that composed human adipose-derived stem cell–laden alginate hydrogel and reinforcing PLA nanofibers ​and human adipose-derived stem cells (hASCs). Their results showed promoted cell proliferation and increased metabolic activity [235]. Other examples include the work by Gruene et al. wherein they demonstrated laser-assisted bioprinting of hASCs mimicking the cell lineage composition of the native adipose tissue [236].

### 4.6 Cancer

Cancer remains one of the most common life-threatening diseases in the world with challenging treatment. Conventional 2D models of cancer tissues cannot closely mimic the native tumor microenvironment. Therefore, there is need to develop 3D cancer tissue models with more physiologically relevant characteristics. Bioprinting technologies offer promising applications in cancer research by forming highly controllable cancer tissue microenvironment. Bioprinted cancer models represent a significant improvement over 2D models by mimicking the complexity of the native tumor tissues [237].

Recapitulation of cancer tumors using 3D bioprinting is a promising approach to test drug efficacy and *in vitro* cancer modeling. For example, King et al. generated an artificial human breast cancer using extrusion bioprinting to simulate the progression of cancer in breast stromal tissues [238]. They used human breast cancer cells and breast stroma cells including adipocytes, mammary fibroblasts, and endothelial cells. The breast cancer and stroma cell aggregates were used as bioinks by self-assembly of the cells as crosslinking mechanism. The aim was to test chemotherapeutic effect of tamoxifen. Higher chemoresistance of cells was observed in the bioprinted tissues compared with those cultured as 2D monolayers. In another work, Zhao et al. used fibrinogen-gelatin-alginate as a bioink with viscosity of about 11 ​Pa ​s at 10°C containing cervical cancer cells [239]. They printed human cervical cancer cells (HeLa cells) within a porous 3D architecture to ensure the oxygen supply to the cells. They used thermal crosslinking for gelatin gel and chemical crosslinking of alginate by CaCl_2_ solution. The chemosensitivity of paclitaxel from HeLa cells in the 3D bioprinted constructs was increased compared with cells in a 2D monolayer. Recently, Langer et al. investigated the 3D bioprinting capability to improve *in vitro* tumor tissue models by using multiple cell types into scaffold-free tumor tissues [240]. They generated tumor tissues from distinct subtypes of breast or pancreatic cancer and showed that this technique can model patient-specific tumors by using primary patient tissue. The intrinsic, extrinsic, and spatial tumorigenic phenotypes in bioprinted tissues were investigated and found that cellular proliferation, ECM deposition, and cellular migration were changed in response to extrinsic signals or therapies. Their findings showed that multiple cell–type bioprinted tissues can mimic aspects of *in vivo* neoplastic tissues and provide a reliable model for the interrogation of multiple tumorigenic endpoints in the context of distinct tumor microenvironments. Huang et al. used DLP-based bioprinting to generate biomimetic tissues with incorporated vasculatures to study effects of geometric cues on migration speed of tumor cells (HeLa cell) and normal fibroblast cells (10T1/2) [241]. They used PEGDA to make constructs because of its tunable mechanical properties and biocompatibility. The embedded vasculatures using three different channel widths (25, 45, and 120 ​μm) to mimic blood vessels of different sizes *in vivo*. Their results showed that HeLa cells migrated at increased speeds in narrower channels, while the fibroblasts migration speed was not affected by the channel width. This work introduced a method to model different responses of cancerous cells and non-cancerous cells to different geometric cues, which could potentially be used as a tool to screen anti-migratory molecules.

There are some limitations for current 3D bioprinted cancer models in terms of cell types and models that closely represent the *in vitro* tumor microenvironment. Future work with utilizing specific cells and primary and patient-specific cancer cells can provide more insights on the progress, diagnosis, and treatment of cancer diseases. In addition, further research is still needed to develop biomaterials and printing technologies to make scaffolds that can mimic the dynamic and biochemical environment of tumors [242].

## 5 Challenges and future outlook

There is advancing activity in the field of developing multimaterial multicellular bioinks as well as in developing necessary printing tools and techniques as discussed here and summarized in Table 2. These developments are however faced with different types of challenges that are related to bioinks, tools, construct development, *in vivo* function, and further translation to industry and clinical practice following regulatory body approval.

**Table 2:** Characteristics of different heterogeneous bioinks for tissue fabrication.

Biomaterial	Cell/soluble factor type	Printing method	Printing conditions	Bioink viscosity	Reference
PCL and decellularized adipose, cartilage, and heart tissues	Human ASCs, hTMSCs, and rat myoblast cells	Extrusion	PCL at 80°C Cell-laden gel at below 15°C	2.8–23.6 ​Pa ​s	[175]
Gelatin type A (10 w/v%), GelMA (10 w/v%), fibrinogen (10 w/v%), and 4-arm PEG amine (20 w/v%)	Human dermal fibroblasts and HUVECs	Extrusion	37°C 1–2.5 ​bar pressure 5 ​mm/s printing speed	Not reported	[87]
Alginate (from 1.0 to 4.0% w/v) and GelMA (4.5% w/v)	HUVECs	Coaxial needle extrusion	Room temperature 1–6 ​mm/s printing speed	0.08 ​Pa ​s	[25]
HA-poly(*N*-isopropylacrylamide) and HA methacrylate (2 ​wt%)	Chondrocytes	Extrusion	Surface temperature between 35°C and 38°C 1.5 ​bar pressure 500 ​mm/min feeding rate	Not reported	[245]
GelMA (5 and 7 ​wt%), alginate (1, 2, and 3 ​wt%), and PEG (1, 2, and 3 ​wt%)	HUVECs and MSCs	Coaxial nozzle extrusion	Room temperature 2–6 ​mm/s printing speed	28–54 ​Pa ​s at different PEG concentrations	[246]
Methacrylated poly(vinyl alcohol) and GelMA	Human endothelial colony forming progenitor cells and human MSCs	DLP	Room temperature	12–14 ​mPa ​s	[203]
PEG (10 ​wt%) and GelMA (5 ​wt%)	NIH 3T3 fibroblasts	Stereolithography	Room temperature	Not reported	[247]
PEG (4% (w/v)) and silk fibroin (up to 1.5% (w/v)) and melanin nanoparticle (up to 1 ​wt%)	NIH 3T3 fibroblasts	DLP	Room temperature	0.01–0.1 (at the shear rates 1-10 ​S^−1^)	[248]
GelMA (10 ​wt%) and PEG (5–20 ​wt%)	MSCs	Stereolithography	Room temperature	Not reported	[191]
Alginate 2%	L929 mouse fibroblasts	Coaxial nozzle extrusion	Room temperature	Not reported	[249]
GelMA and gellan gum	MSCs	Extrusion	475 ​mm/min, at room temperature	Not reported	[91]
Polyurethane	Neural stem cells	Fused deposition manufacturing	37°C	Not reported	[250]
GelMA 5% (w/v)	HUVECs and 10T1/2	DLP	Room temperature	Not reported	[200]
Nanocellulose and alginate/HA	iPSC	Extrusion	Room Temperature 10–20 ​mm/s feed rate 20–30 ​kPa pressure	1–1000 ​Pa ​S at 1 ​S^−1^ depending on the concentration	[251]
Pluronic diacrylate	Bovine chondrocyte	Extrusion	Room temperature 80 ​mm/min Pressure of 2 Bar	∼35 ​mPa ​s	[252]
PCL reinforced hydrogels (agarose, alginate, GelMA and BioINK™)	MSCs	Extrusion	21–37 ​°C (70 ​°C for printing PCL) 0.06–0.2 ​MPa	Not reported	[253]
Methacrylated HA	MSCs and BMP-2	Stereolithography	Room temperature	Loss modulus ∼7–22 ​Pa	[71]
GelMA/alginate 1.0%	HepG2 and HUVECs	Laser assisted extrusion	500 ​μl ​min^−1^ and 500 ​mm ​min^−1^	Not reported	[76]
20 ​mg/ml fibrinogen, 30 ​mg/ml gelatin, 20 ​μg/ml aprotinin, 10% glycerol, and 3 ​mg/ml HA	Rat ventricular cardiomyocytes	Multimaterial nozzle extrusion	PCL: PCL frame was printed at 98°C at 750 ​kPa pneumatic pressure Sacrificial hydrogel printed at 18°C at 100 ​kPa pneumatic pressure 100 ​mm/min	Not reported	[254]
GelMA, chondroitin sulphate amino ethyl methacrylate, and methacrylated HA	Bone marrow-derived human MSCs	Coaxial extrusion printing	Room temperature	∼1 ​Pa ​s to 300 ​mPa ​s	[255]
PEG-alginate-nanoclay 2.5–5%	Human MSCs	Extrusion	Room temperature	10–800 ​Pa ​s (at the shear rates 1–10 ​S^−1^)	[256]

PEG, polyethylene glycol; MSC, mesenchymal stem cells; GelMA, gelatin methacryloyl; HA, hyaluronan; PCL, poly(ε-caprolactone); HUVECs, Human umbilical vein endothelial cells; DLP, digital light processing; hTMSC, human turbinate mesenchymal stromal cells.

Challenges related to multicomponent bioinks include the development of appropriate materials having shear-thinning properties with cell-friendly property and other desired biological characteristics for different tissue engineering applications. Unfortunately, most of hydrogels that have been used for 3D bioprinting of heterogeneous and biomimetic structures are degraded relatively fast and loose their structure and in 2 to 3 weeks. Although various approaches such as the use of reinforcing fibers [235], [243] or particles [90], [91], [244] have been proposed to address this problem, the problem still remains unresolved to satisfactory level to have reliable.

Constructs that can be used in clinic especially tissues and organs, such as musculoskeletal, cardiovascular, and renal tissues which are exposed to high mechanical storage and load are highly demanding. Of course, another important application of 3D bioprinted tissue constructs is as disease tissue models for drug development studies. However, engineered tissues need to be more biomimetic and sustain their structure and function for longer periods of time even for this *in vitro* application. Therefore, new materials need to be developed for biofabrication of biological tissues using bioprinting technology.

Advanced bioprinting technologies should be developed to obtain large-scale tissue constructs in a rapid and high-throughput manner. Scaling up of bioprinting process is another challenge to enable the transfer of technology to wider industrial production. For this purpose, the speed of bioprinting was recently addressed, and new bioprinters were developed. The challenge still remains to fabricate more combinations of materials and cells, which are today limited to few. The other research avenue would be the transfer of technology to the operating room where bioprinting can be individualized to bioprint tailored structures and controlled by surgeon, such as hand-held bioprinting [257]. Robot-controlled bioprinters can be further a part of surgical team and dynamic tools in future hospitals. The constructs will be smart, dynamic, reactive, and communicative [45], [257]. Minimally invasive administration of miniaturized bioprinted constructs will be a further addition to achieve faster recovery and reduced complications.

The other challenge is related to defining balance between different concentrations of materials, cells, and biomolecules to achieve optimal results in tissue regeneration and remodeling. So far, most of studies took place *in vitro* [4], [258], [259]. While the ultimate challenge of *in vitro* studies is to screen the success of 3D bioprinting, such heterogeneous constructs should be tested and validated *in vivo*. Fabricated heterogeneous and biomimetic tissues should then be implanted in small and large animal models to evaluate tissue function in an acute and chronic manner.

The use of materials that have special properties, such as smart materials with stimuli-responsive capabilities of shape memory represent another underexplored but potential area for development of more biomimetic and dynamic tissue implants that can contribute the future of health care. Such smart and custom-designed implants can be delivered to the body in the least invasive way [45]. Implanted constructs, whether made of conventional or smart materials need to survive, integrate, and remodel in the body. In this regard, a major challenge remains the vascularization of constructs. Various methods of using angiogenic factors or cells have been explored, and the augmentation of engineered constructs with microsurgical flaps can also provide a viable solution [260]. In addition, an important benefit of multicellular and multimaterial bioprinting is to build in vessels that are capable of anastomosing with host vessels following implantation. Once this step is accomplished, one may also think about including nervous and lymphatic components to the constructs in future. Moreover, cell microenvironment dynamically interacts with the cells. The ECM stiffness is known to be an important indicative of the cell phenotype. This implies the need for dynamic hydrogels with tunable viscoelastic and time dependent behavior [261]. Adaptive crosslinking in hydrogels with reversible bonds that are responsive to mechanical deformations can support complex cell activities and long-term cell function [34]. Hence, more attention should be paid to uncover capabilities in programming the cell behavior, controlling the cell spreading ​and matrix mechanics [262].

Regulatory issues for the approval of 3D bioprinted constructs remain another important challenge that needs to be addressed properly. Although bioprinted constructs were produced from the same materials that are clinically used as implants, 3D bioprinted implants need to go through comprehensive evaluation prior to their use in clinic. This led to delay in wide applications of bioprinted constructs in clinic. So far, the application of 3D implants in the clinic remains limited to few sporadic cases. It has to be noted, however that these regulations differ from one country to another. In some countries, 3D implants made from the same materials used in previously approved materials seem to be allowed [263]. Wider discussion and common consensus need to be developed to help regulatory bodies to come up with appropriate regulations and thereby enhance industrial production and clinical application of bioprinted constructs.

Important future directions in 3D bioprinting include the production of more dynamic and complex structures than can be a step closer to mimic the native tissues. Using advances made in these areas, stem cells can be induced to differentiate using biological cues in the bioink itself. Moreover, various chemical, electrical, and mechanical stimuli can be incorporated in bioinks to differentiate stem cells to desired multitude of lineages that are required to constitute biological tissues. Supportive cells such as fibroblasts or glial cells can also be used, and autocrine function of combined cells in the bioink and resulting bioprinted constructs can be harnessed. However, the relationship between parenchymal, supportive, vascular, and neuroendocrine elements have to be precisely defined [264] leading to further development of the resulting tissue-like constructs.

## 6 Conclusions

Advanced 3D bioprinting techniques and multicomponent bioinks have recently been developed to mimic the structure of the native tissues. Currently developed multicomponent bioinks are composed of natural, synthetic, or hybrid natural-synthetic biomaterials, different types of cells, and soluble factors. Moreover, some nanobiomaterials can be added to such bioinks to mimic the structure and function of the native tissues. Advanced bioprinting technologies have enabled us to bioprint multimaterial and multicomponent bioinks with spatial and microscale resolution in a rapid and continuous manner, aiming to reproduce the complex architecture of the native tissues. This work reviewed important advances in multicomponent bioinks and bioprinting technologies to fabricate biomimetic tissue constructs. There still remains important major challenges that need to be addressed to enable the translation of the technology to the clinic. It is hoped that multicomponent bioinks and technologies greatly advance the field of biomimetic tissue engineering for therapeutic and pharmaceutical applications.

## Conflict of interest

The authors declare that they have no known competing financial interests or personal relationships that could have appeared to influence the work reported in this paper.
